# Enhancer reprogramming promotes the activation of cancer-associated fibroblasts and breast cancer metastasis

**DOI:** 10.7150/thno.75853

**Published:** 2022-10-24

**Authors:** Qian Li, Xuejiao Lv, Chunyong Han, Yu Kong, Zhongye Dai, Dawei Huo, Ting Li, Dapeng Li, Wei Li, Xing Wang, Qian Zhao, Jie Ming, Wen Yang, Yang Chen, Xudong Wu

**Affiliations:** 1 State Key Laboratory of Experimental Hematology, The Province and Ministry Co-sponsored Collaborative Innovation Center for Medical Epigenetics, Key Laboratory of Immune Microenvironment and Disease (Ministry of Education), Department of Cell Biology, School of Basic Medical Sciences, Tianjin Medical University, Tianjin 300070, China.; 2 Department of Breast Reconstruction, Tianjin Medical University Cancer Institute and Hospital, Key Laboratory of Breast Cancer Prevention and Therapy, Tianjin Medical University, Ministry of Education, Tianjin 300060, China.; 3 National Clinical Research Center for Cancer, Key Laboratory of Cancer Prevention and Treatment of Tianjin, Tianjin Clinical Research Center for Cancer.; 4 Department of Thyroid and Neck Tumor, Tianjin Medical University Cancer Institute and Hospital, Tianjin 300060, China.; 5 Department of Pathology, Tianjin First Center Hospital, Tianjin 300192, China.; 6 Department of Breast and Thyroid Surgery, Union Hospital, Huazhong University of Science and Technology, Wuhan, Hubei Province, 430022, China.; 7 The State Key Laboratory of Medical Molecular Biology, Institute of Basic Medical Sciences, Chinese Academy of Medical Sciences and School of Basic Medicine, Peking Union Medical College, 5 Dong Dan San Tiao, 100005, Beijing, China.; 8 Department of Neurosurgery, Tianjin Medical University General Hospital, Tianjin 300052, China.

**Keywords:** cancer-associated fibroblasts, metastasis, enhancers, transcription factors, JUN

## Abstract

**Rationale:** Cancer associated fibroblasts (CAFs) are a subpopulation of cells within the tumor microenvironment that usually promote cancer progression and metastasis. Hence it is critical to find out the driving factors and mechanisms for the development of CAFs from normal fibroblasts (NFs) in response to sustained stimulation of cancer cells. Here we perform transcriptomic and epigenomic analyses in paired NFs and CAFs associated with breast cancer metastasis to investigate the molecular mechanisms for stromal fibroblasts reprogramming.

**Methods:** We conducted transcriptomic analyses in paired NFs and CAFs isolated from clinical specimens of breast cancer patients with metastasis. Meanwhile, genome-wide mapping of histone marks H3K4me1 and H3K27ac was also performed to characterize CAF-specific enhancer landscape. The function and mechanisms of activated JUN in stromal fibroblasts were studied using* in vitro* and *in vivo* models.

**Results:** We have identified CAF-specific signature genes and activated enhancers, which are significantly associated with pro-metastatic programs. Among the CAF activated enhancers, FOS and JUN family of transcription factors are enriched. In line with this, we find that JUN protein is highly activated in the stroma of metastatic breast cancers. And through gain and loss-of-function studies, we demonstrate that activated JUN is necessary and sufficient to remodel enhancers and maintain the activation of CAF-specific enhancers, and thereby promotes breast cancer invasiveness in a non-cell-autonomous manner.

**Conclusions:** Our study gets an insight into the transcriptomic features of invasive breast stroma and transcription regulatory mechanisms for stroma cell transformation, providing a proof-of-concept of stroma-targeting strategy in cancer treatment.

## Introduction

It has been well recognized that tumor initiation and progression are controlled by both cell-autonomous and non-cell-autonomous mechanisms. During tumor development or in response to therapeutics, fibroblasts, immune cells, the extracellular matrix and other cellular or non-cellular components constitute a highly complex and plastic tumor stroma. The specialized niche, also termed as tumor microenvironment (TME) is reconstructed by cancer cells for their own benefit 1, 2. In some cases, the gene expression of stromal cells even defines the cancer subtypes with metastasis and relapse 3-5. Accordingly, suppression of stromal functions leads to the constraint of tumor progression 1, 3, 6-8. Therefore, mechanistic understanding of TME formation and adaptation to cancer progression may open new anti-stroma therapeutic avenues.

Breast cancers are the commonest women malignancy and the second leading cause of cancer-related death in women in the world. Different molecular subtypes have been identified. The luminal subtypes (A or B) account for the majority of breast cancers and are characterized by expression of hormone receptors for estrogen (estrogen receptor α, ERα) and/or progesterone (PR) with or without expression of human epidermal growth factor receptor 2 (HER2). Whereas basal-like subtypes do not express hormone receptors and have the highest recurrence rate. Irrespective to the subtypes, the metastasis to the lymph nodes and distant organs, such as bone, lung, liver, and brain is associated with unfavorable prognosis 9. Thus, it is urgent to dissect molecular mechanisms for breast cancer metastasis.

Notably breast cancers are characterized by an extensive desmoplastic stroma, mainly composed by cancer associated fibroblasts (CAFs). Compared with normal fibroblasts (NFs), CAFs generally have a higher proliferation rate and are more invasive. More importantly, CAFs have been shown to potentiate metastasis by releasing multiple growth factors and cytokines, and remodeling the extracellular matrix, etc. Inhibition of distinct pro-metastatic factors in subtype-specific CAFs has been shown to counter breast cancer progression 5, 10-14. However, a common molecular basis for the activation of pro-metastatic programs is still lacking.

Emerging evidences have demonstrated that CAFs are activated fibroblasts as a result of cancer cells' education 10, 15. A variety of extracellular signaling pathways have been found to drive the transition from NFs to CAFs (CAF activation) 14, 16-21. In contrast, how intracellular mechanisms are co-opted for the reprogramming of cell fates remains poorly characterized. Different from the genome instability in epithelial cancer cells, the genome in CAFs remains relatively stable during tumor progression. The differences between NFs and CAFs in the phenotypes or expression profiles mainly originate from epigenomic alterations. Therefore, a systematic view of the dynamic changes of chromatin landscapes during CAF activation is the key to understanding the molecular mechanisms for the stroma cell transformation.

Among the *cis*-regulatory elements, enhancers play key roles in the control of the specific gene expression programs over large distance that determine cell types or cellular states 22-24. The recruitment of lineage-defining or signal-regulated transcription factors (TFs) on the emerging enhancers is an essential targeting process for the intervention of cell fates in response to intracellular or extracellular cues. Meanwhile chromatin-modifying enzymes are deposited to catalyze histone H3 lysine 4 mono-methylation (H3K4me1), and additionally catalyze histone H3 lysine 27 acetylation (H3K27ac) upon activation 25. Enhancer reprogramming has been shown to be a leading cause of changes in cell types or functions 26, 27. Therefore, it would be interesting to find out how enhancers are remodeled during CAF activation.

Here by genome-wide mapping of the characteristic histone marks H3K4me1 and H3K27ac in paired NFs and CAFs derived from metastatic breast cancer patients, we identify the breast CAF-specific active enhancers (*cis*) and potential binding TFs (*trans*) that may drive CAF activation. Among the top enriched binding factors are FOS and JUN family proteins, which usually form activator protein 1 (AP-1) dimmers and bind to their regulatory elements. And we verify the crucial roles of activated AP-1 regulatory networks (*cis* and *trans*) in NFs to induce CAF-activated enhancers and acquire pro-metastatic functions. More importantly, we demonstrate that the inhibition of JUN transcriptional activity disrupts the AP-1 regulatory networks in CAFs and attenuates breast cancer metastasis* in vitro* and *in vivo*. Therefore, our study deciphers the dynamic chromatin landscape and transcriptional network during CAF activation, which will hopefully provide a promising strategy for anti-cancer treatments.

## Results

### Pro-metastatic programs are activated in metastasis associated CAFs

To date, rather few datasets are available to compare gene expression profiles of stromal cells from either normal tissues or breast cancers. Here we isolated pairs of CAFs and NFs respectively from dissected tumor tissues or corresponding para-tumoral non-malignant tissues of several patients with breast cancers. To identify the potential transcriptional signatures associated with invasiveness, we selected the cases with lymph node (LN) metastasis, regardless of breast cancer subtypes (Figure S1A). To avoid influence by therapeutics, none of the patients have received radiotherapy or chemotherapy before surgery (Table S1).

**Figure 1:**
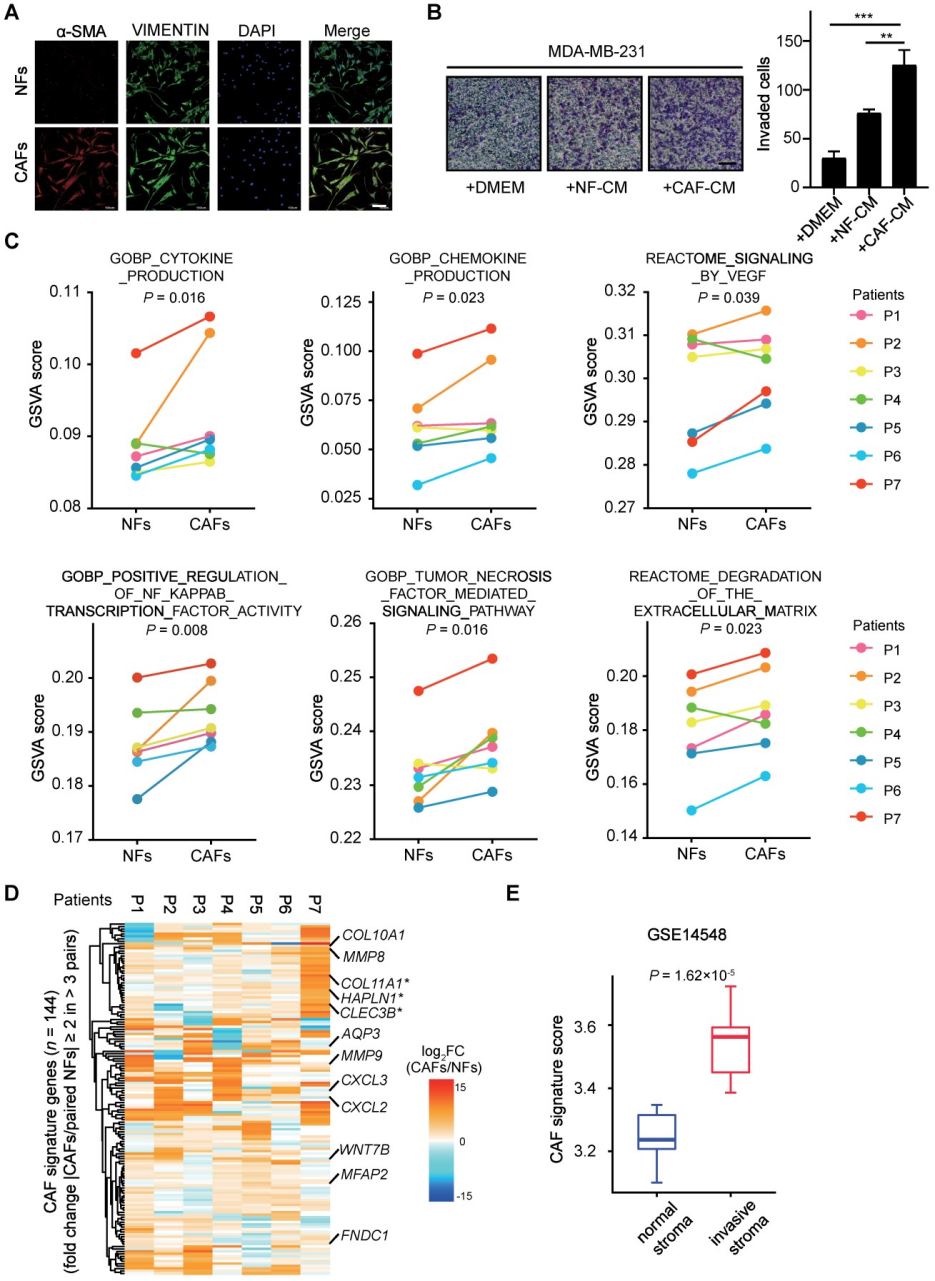
** Transcriptional alterations in metastasis associated CAFs.** (**A**) α-SMA (red), VIMENTIN (green) and 4′,6-diamidino-2-phenylindole (DAPI) (blue) staining of NFs and matched CAFs isolated from breast cancer patients. Scale bars, 100 μm. (**B**) Transwell assays showing invaded MDA-MB-231 after incubation with DMEM, NF-conditional medium (CM), CAF-CM for 48 h. Left: representative images are shown. Scale bars, 100 μm. Right: quantification analysis of invaded cells is shown. Bars represent mean ± SD (standard deviation), *n* = 3. *P* value was determined by two-sided unpaired *t* test. **, *P* < 0.01; ***, *P* < 0.001. (**C**) GSVA analyses for paired RNA-seq data (NFs/CAFs) from seven patients. Line plot comparisons of GSVA scores of indicated gene sets from Molecular Signature Database (MsigDB) are shown. *n* = 7 biologically independent patient samples. P1-P7, patient 1-7 respectively. *P* values were determined by one-sided Wilcoxon signed rank exact test. (**D**) CAF signature genes consisting of 144 genes were identified (fold change (CAFs/paired NFs) ≥ 2 in more than 3 pairs). Heatmap shows log_2_ transformed fold change (CAFs/paired NFs) in mRNA levels of CAF signature genes, using RNA-seq data in seven pairs of NFs and CAFs. Several reported tumor-promoting genes are labeled. Asterisks (*) mark genes known to encode CAFs' secreted factors. (**E**) Boxplot shows CAF signature score in normal stroma and invasive stroma for GSE14548 microarray dataset. The lower and upper hinges represent the first and third quartiles. The midline represents the median. The upper and lower whiskers extend from the hinge up to 1.5 × IQR (inter-quartile range). CAF signature score was calculated as mean mRNA levels of CAF signature genes. *P* value was determined by two-sided unpaired *t*-test.

As previously characterized, NFs and CAFs are both positive for fibroblast marker VIMENTIN, whereas CAFs specifically exhibit a high expression level of α-smooth muscle actin (α-SMA) (Figure 1A). To examine their effects on cancer cells, we performed transwell assays with breast cancer cells. MDA-MB-231 cells were incubated with conditional medium (CM) from either NFs or CAFs after *in vitro* expansion for 3~5 passages. Compared with the mock medium or the CM from NFs, the CM from CAFs significantly promotes breast cancer cell invasion ability (Figure 1B). These data indicate that the isolated CAFs maintain their intrinsic pro-metastatic activity.

To get an insight into the mechanisms for CAF-supported metastasis in breast cancers, we performed RNA-sequencing (RNA-seq) analysis with seven pairs of primary NFs and CAFs within five passages of optimal culture. As shown by the line plots of Gene set variation analysis (GSVA) 28 score, a significantly upregulated expression of pro-metastatic genes enriched with cytokine production, chemokine production, signaling by VEGF, positive regulation of NF-κB transcription factor activity, tumor necrosis factor mediated signaling pathway, degradation of the extracellular matrix 29 was observed in CAFs (Figure 1C). Nevertheless, the altered expression of individual genes in each of gene sets vary among distinct patient samples, in reflection of inter-tumoral stromal heterogeneity (Figure S1B-G).

To identify the common features, we identified CAF signature genes composing of 144 genes with fold change (paired CAFs/NFs) of mRNA expression levels ≥ 2 in more than 3 pairs (Figure 1D). Despite apparent differences among individual samples, several of these primary CAFs showed similar patterns of CAF signature genes (Figure S1H). A number of genes such as *COL11A1* 30, *HAPLN1* 31 and *CLEC3B* 32 with reported tumor-promoting functions in stroma were among the list of signature genes. And according to the dataset from TCGA, their high expression levels are significantly correlated with lower overall survival probability of breast cancer patients (Figure 1D, Figure S1I). Taking advantage of another independent published microarray dataset (GSE14548), we compared the expression levels of these CAF signature genes in normal and invasive stroma. Interestingly, the CAF signature genes showed significantly higher expression in invasive stroma than the normal counterparts (Figure 1E). Thus, we have successfully generated a transcriptome dataset of breast cancer stroma, which clearly shows that the deregulated expression profiles in CAFs are associated with cancer metastasis and unfavorable prognosis.

### Enhancer reprogramming accompanies with CAF activation

**Figure 2:**
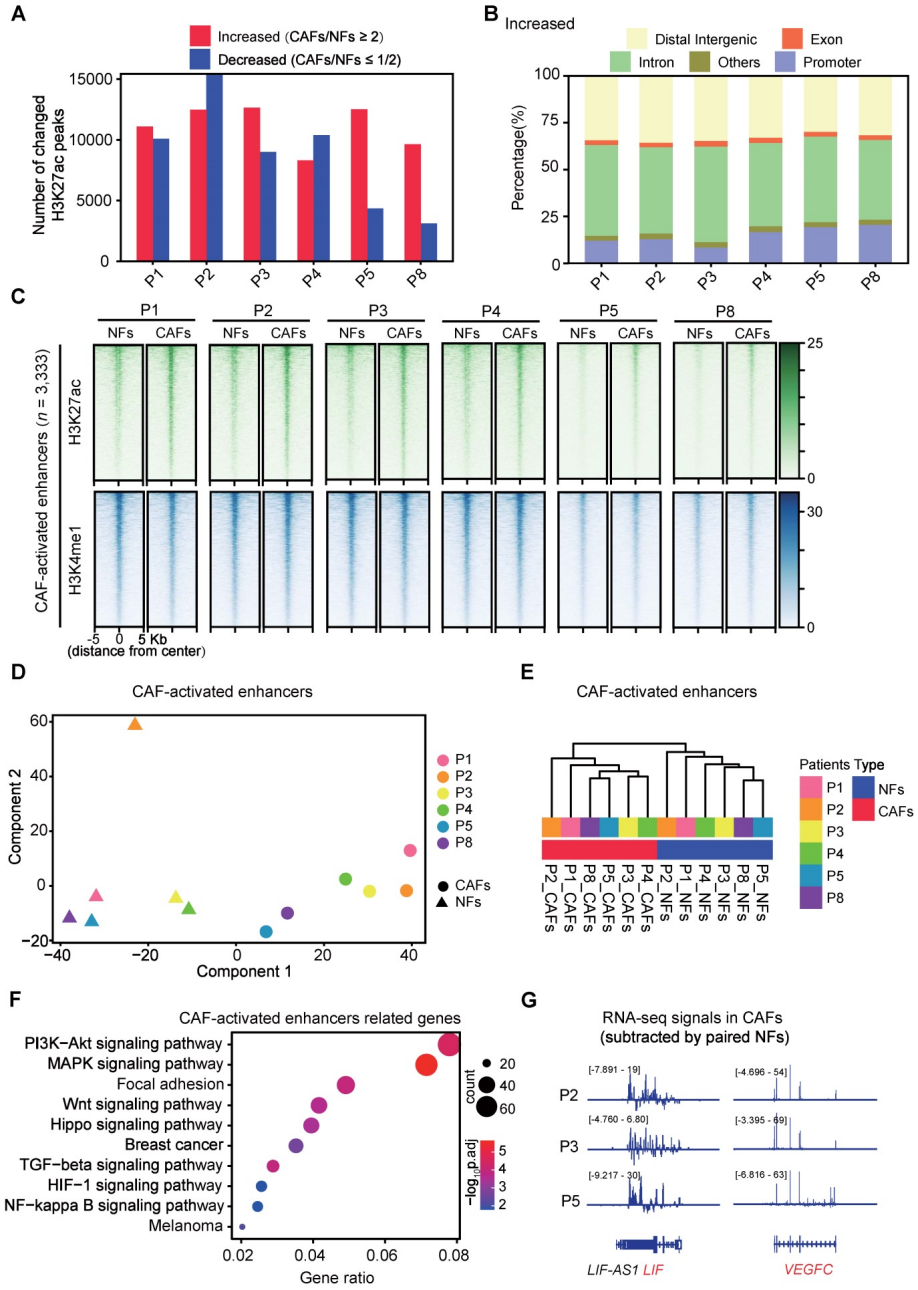
** Enhancer reprogramming accompanies with CAF activation.** (**A**) The number of peaks with increased H3K27ac (≥ 2-fold than paired NFs) and decreased H3K27ac (≤ 1/2-fold than paired NFs) are shown. P1-P5, patient 1-5; P8, patient 8. (**B**) Genomic distribution of peaks with increased H3K27ac enrichment. (**C**) Heatmaps of H3K27ac and H3K4me1 CUT&Tag-seq signals in six pairs of NFs and paired CAFs across regions of ± 5,000 bp surrounding the center of CAF-activated enhancers. (**D**) PCA plot of CAF-activated enhancers for H3K27ac signals in each sample. The circle represents CAF samples while the triangle represents NF samples. (**E**) Unsupervised hierarchical clustering of CAF-activated enhancers for H3K27ac signals in each sample. (**F**) Highly enriched KEGG pathways of nearest genes of CAF-activated enhancers are shown. (**G**) Tracks of RNA-seq signals of designated genes in CAFs subtracted by paired NFs.

To understand the driving mechanisms for CAF activation, we performed H3K27ac Cleavage Under Targets and Tagmentation (CUT&Tag) 33 analysis in six pairs of NFs and CAFs that were successfully expanded for a few passages (Table S2). Despite similar genomic distribution across these fibroblasts (Figure S2A), dramatic changes of H3K27ac enrichment were observed in all paired samples. Thousands of peaks were specifically identified either in CAFs or in NFs (|fold change| ≥ 2) (Figure 2A). Majority of these peaks were localized outside of annotated promoters (Figure 2B, Figure S2B). After identifying TOP 2,000 promoter regions and non-promoter regions with increased H3K27ac enrichment in CAFs compared with paired NFs, we conducted Principal Component Analysis (PCA) for six paired samples. NFs and CAFs exhibit a clear separation at non-promoter regions, although they are distinguishable at both promoter and non-promoter regions (Figure S2C-D). Moreover, Kyoto Encyclopedia of Genes and Genomes (KEGG) analysis showed that the nearest genes of TOP 2,000 non-promoter regions are highly enriched for cancer related signaling pathways (Figure S2E). Hence these regions with increased H3K27ac enrichment represent potential enhancers for CAFs.

To identify activated enhancers in CAFs, H3K4me1 CUT&Tag analyses were performed in six pairs of NFs and CAFs in addition to H3K27ac (Table S2). At H3K4me1+ sites, we identified 3,333 regions with H3K27ac levels in CAFs ≥ 2-fold than the one in paired NFs in more than 3 pairs, and defined them as the CAF-activated enhancers. At these regions, H3K4me1 profiles look highly similar whereas H3K27ac enrichment is dramatically altered in all paired NFs and CAFs, suggesting that H3K4me1 is preset in NFs and maintained during CAF activation (Figure 2C, Figure S3A-B).

And these activated regions in most of the CAF samples are significantly correlated with higher expression levels of their associated genes compared with their normal counterparts, despite transcriptomic heterogeneity among samples (Figure S3C). More importantly, PCA analysis showed pretty clear separation of CAF-activated enhancers among all NFs and CAFs (Figure 2D). This was further supported by unsupervised hierarchical clustering analysis (Figure 2E). Similarly, we identified 1,881 regions with comparable H3K4me1 enrichment and decreased H3K27ac signals (≤ 1/2-fold than paired NFs in more than 3 pairs) in CAFs (Figure S4A) as the CAF-repressed enhancers. Though CAFs are also distinguishable from NFs according to PCA Analysis of the repressed enhancers, no significant difference in mRNA levels of associated genes was observed in any pair of NFs and CAFs (Figure S4B-C). In contrast, KEGG analysis revealed that the CAF-activated enhancer-associated genes are significantly enriched for signaling pathways strongly related to focal adhesion, paracrine signaling and cancer progression (Figure 2F). For instance, the genomic regions of secreted factor-coding genes such as leukemia inhibitory factor (*LIF*), vascular endothelial growth factor C (*VEGFC*), interleukin 1 beta (*IL1B*) and fibroblast growth factor 1 (*FGF1*), show typical chromatin features of the CAF-activated enhancer (Figure S5). Accompanied with higher H3K27ac enrichment, higher expression levels of these pro-metastatic genes were observed in primary CAFs than in paired NFs (Figure 2G). Collectively, these data indicate that enhancer reprogramming is significantly associated with CAF activation.

### CAF-activated enhancer-enriched JUN is activated in tumor stroma

**Figure 3:**
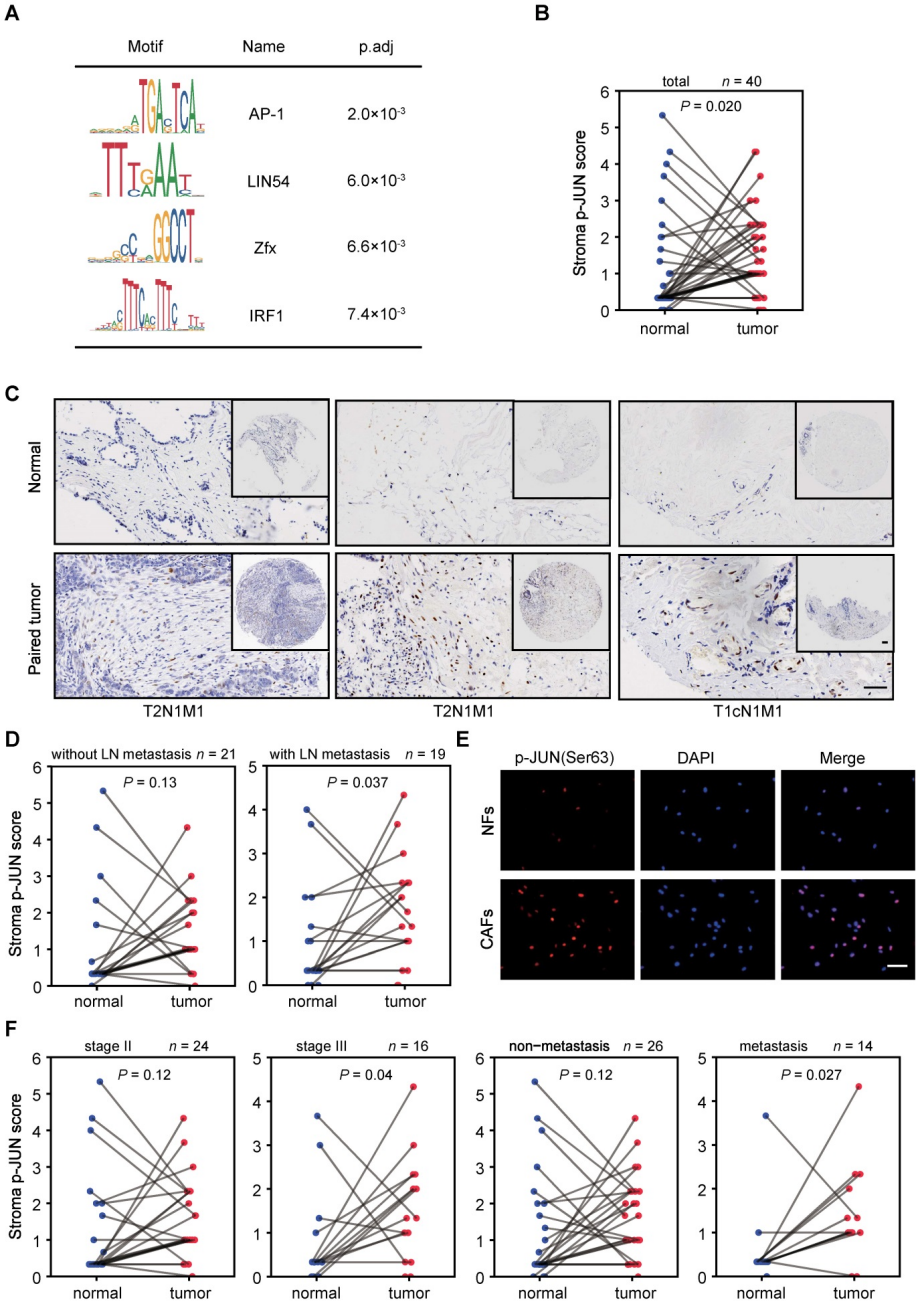
** Identification and characterization of JUN in breast cancer stroma**. (**A**) Motif analysis of regions of ± 300 bp surrounding the center of CAF-activated enhancers using MEME Suite. (**B**, **D** and **F**) Stroma immunostaining scores of phosphorylated JUN (p-JUN) in para-cancerous and paired tumor tissues of indicated conditions are shown as line plot. The numbers of samples for each condition are marked above the panel. *P* value was determined by one-sided paired *t* test. (**C**) Representative images of p-JUN immunostaining in three pairs of para-cancerous tissues and paired breast cancer tissues. Clinical TNM stage was labeled at the bottom. T, tumor. N, node. M, metastasis. Scale bars, 100 μm. (**E**) Immunostaining of p-JUN (red) and DAPI (blue) in NFs and paired CAFs. Scale bars, 100 μm.

To determine the potential driving TFs for the enhancer reprogramming during CAF activation, we performed TF binding motif searches at the CAF-activated enhancers. AP-1 motif enrichment is ranked first (Figure 3A) using MEME Suite 34. Besides, Hypergeometric Optimization of Motif EnRichment (HOMER) software 35 also identified AP-1 binding motif as one of the most significantly enriched sequences (Figure S6A). AP-1 family proteins, well characterized homodimers or heterodimers of TFs which consist of JUN (JUN, JUND, JUNB) and FOS (FOS, FOSB, FOSL1, FOSL2) family members, are known to be involved in cell proliferation, apoptosis and neoplastic transformation 36. Recent studies have supported that AP-1 play critical roles in selection and formation of cell-type specific enhancers 37-40. Because JUN is a common component of JUN/JUN homodimers and JUN/FOS heterodimers 36, we focused to examine JUN expression in breast cancer stroma.

According to RNA-seq or immunofluorescence (IF) analysis, no significant changes of JUN mRNA or protein levels were observed in the isolated NFs and CAFs (Figure S6B-C). Nevertheless, when we performed immunohistochemical (IHC) analysis in tumor microarrays, we found that JUN protein levels are significantly higher in tumor stroma than paired para-cancerous stroma in all of the tested samples regardless of their TNM stages or metastasis states (Figure S6D). It is likely that the high JUN expression levels are not perfectly maintained in the* in vitro* culture condition.

Though AP-1 activity is strongly induced in response to numerous signals, JUN transcription activity is mainly enhanced by Jun N-terminal kinases (JNK)-mediated phosphorylation at Ser63/Ser73, which is located in the transactivation domain of JUN 41-44. Thus, we followed to compare the levels of phosphorylated JUN (p-JUN). As shown by IHC analysis in tissue microarrays, p-JUN levels are significantly higher in breast cancer stroma, compared to corresponding para-cancerous tissues (Figure 3B, C). It is worth noting that significantly stronger stroma immunostaining of p-JUN is specific in tumors with LN metastasis (Figure 3D). This was also supported by the observation in primary cultured NFs and paired CAFs which were derived from LN-metastasis breast cancer patients (Figure 3E). Interestingly, we also detected significantly higher levels of p-JUN in the stroma of tumors with higher grade or distant metastasis (Figure 3F). Thus, JUN phosphorylation are associated with activated stroma and tumor invasiveness.

### Phosphorylated JUN drives enhancer activation and confers inflammatory CAF-like functions to fibroblasts

**Figure 4:**
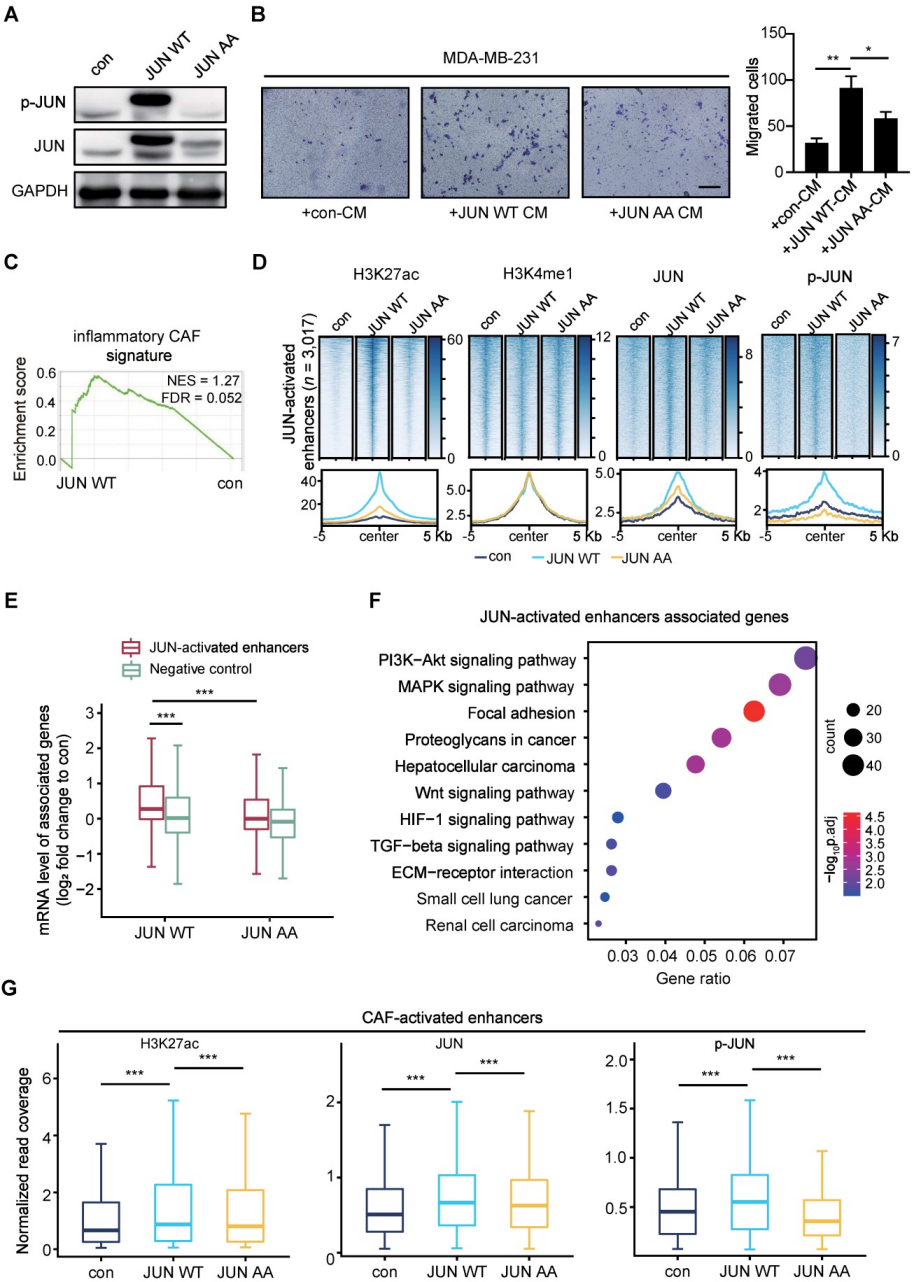
** Phosphorylated JUN induces enhancer activation in fibroblasts and thereby increases cancer cell invasiveness.** (**A**) WB assays showing the levels of p-JUN and total JUN. GAPDH is used as a loading control. (**B**) Transwell assays showing migrated MDA-MB-231 after incubation with CM of MRC5 overexpressing JUN WT, JUN AA or control for 48 h. Left: representative images are shown. Scale bars, 200 μm. Right: quantification analysis of migrated cells is shown. Bars represent mean ± SD (standard deviation), *n* = 3. *P* value was determined by two-sided unpaired *t* test. *, *P* < 0.05; **, *P* < 0.01. (**C**) Gene set enrichment analysis (GSEA) for inflammatory CAF signature based on the RNA-seq data of MRC5 overexpressing JUN WT and control. (**D**) Heatmaps and average profiles of H3K27ac, H3K4me1, JUN and p-JUN ChIP-seq signals across regions of ± 5,000 bp surrounding JUN-activated enhancers center. (**E**) Boxplot shows mRNA level fold change (each designated group (JUN WT or JUN AA) relative to control) of associated genes with JUN-activated enhancers and non-activated enhancers (Negative control). *P* value of comparison the first box with the second box was determined by two-sided unpaired *t* test. *P* value of comparison the first box with the third box was determined by two-sided paired *t* test. ***, *P* < 0.001. (**F**) Highly enriched KEGG pathways of nearest genes of JUN-activated enhancers are shown. (**G**) Boxplots to compare the H3K27ac, JUN and p-JUN ChIP-seq signals at CAF-activated enhancers in each designated group of cells. *P* value was determined by two-sided paired *t* test. ***, *P* < 0.001.

Then we would follow to find out whether phosphorylated JUN is sufficient to confer a CAF-like feature if overexpressed in NFs. Owing to the limited cell numbers and passages of primary fibroblasts, we transduced immortalized human embryonic lung fibroblasts MRC5 cells with FLAG-tagged JUN wild-type (WT) or inactive mutant whose JNK-phosphorylation sites are mutated to Alanine (both Ser63 and Ser73 to As, termed as JUN AA). As examined by Western blot (WB) assays, JUN WT overexpression leads to strikingly higher expression levels of both phosphorylated and total JUN than enforced expression of JUN AA (Figure 4A). It indicates that JUN WT rather than AA may bind to the endogenous promoter and activate its own expression.

To compare their effects on cancer cells, we performed transwell assays with breast cancer cells. MDA-MB-231 cells were incubated with CM from control or JUN WT or AA overexpressed MRC5 cells for 48 h. As shown in Figure 4B, incubation with the CM from JUN WT rather than AA overexpressed MRC5 cells significantly strengthened the migration abilities of MDA-MB-231 cells. To get a further insight into the functional outcome after overexpressing JUN, we performed Gene Set Enrichment Analysis (GSEA) 45, 46 based on the RNA-seq data. Compared to the control group, JUN WT overexpression group exhibited significant enrichment of inflammatory CAF-related signatures, such as chemokine receptors bind chemokines, cytokines and inflammatory response, as well as IL-1 signaling pathway, but not myofibroblastic CAF signature 17, 47, 48 (Figure 4C and Figure S7A). These data confirm that activated JUN is sufficient to induce inflammatory CAF-like functions in fibroblasts.

To further dissect whether and how activated JUN reconfigures the chromatin landscape, we performed Chromatin Immunoprecipitation (ChIP)-seq analyses for H3K4me1 and H3K27ac respectively in control and the two overexpressed MRC5 cells.

Based on the significant increase of H3K27ac levels at H3K4me1+ sites, we identified 3,017 JUN-activated enhancers in JUN WT cells. In contrast, JUN AA fails to significantly induce H3K27ac accumulation at these regions (Figure 4D and Figure S7B). Similar to the CAF/NF comparison, H3K4me1 enrichment remains largely unaltered by either JUN WT or AA on these regions (Figure 4D). These data suggest that the activation of a subset of primed or poised enhancers is specifically driven by JUN WT.

To confirm these enhancers are directly driven by JUN, we also performed ChIP-seq analyses for JUN and p-JUN. As shown in Figure 4D and Figure S7B, enforced expression of JUN WT, but not JUN AA, led to dramatically higher occupancy of total JUN and p-JUN at the JUN-activated enhancers than the control. These data indicate that JUN, especially phosphorylated JUN induces enhancer activation.

We next examined the effects of phosphorylated JUN-mediated enhancer activation on gene expression. As shown in Figure 4E, the expression levels of genes associated with the JUN-activated enhancers are significantly upregulated in JUN WT cells rather than in JUN AA cells, compared with the control cells. For instance, the upregulation of *VEGFC* and *IL1B* expression levels was specifically observed in JUN WT cells (Figure S7C). In contrast, JUN WT does not significantly affect the expression of genes associated with non-activated enhancers (Figure 4E). And KEGG analysis showed that these JUN-activated enhancers-associated genes were significantly enriched for PI3K-AKT and WNT signaling pathway, etc, which are well characterized to be involved in tumor progression (Figure 4F). These enriched signaling pathways are highly consistent with those of CAF-activated enhancers-associated genes (Figure 2F), although the two gene sets only partially overlap (Figure S7D). More importantly, we also detected significantly higher enrichment of H3K27ac, JUN and p-JUN at the CAF-activated enhancers in JUN WT group rather than in JUN AA, compared with the control (Figure 4G, Figure S7E). These findings further support that activated JUN accounts for the activation of CAF-activated enhancers in fibroblasts and thereby the enhancer remodeling and altered gene expression underlie the CAF-like functions.

### Phosphorylated JUN is required for maintenance of the CAF-activated enhancers

**Figure 5:**
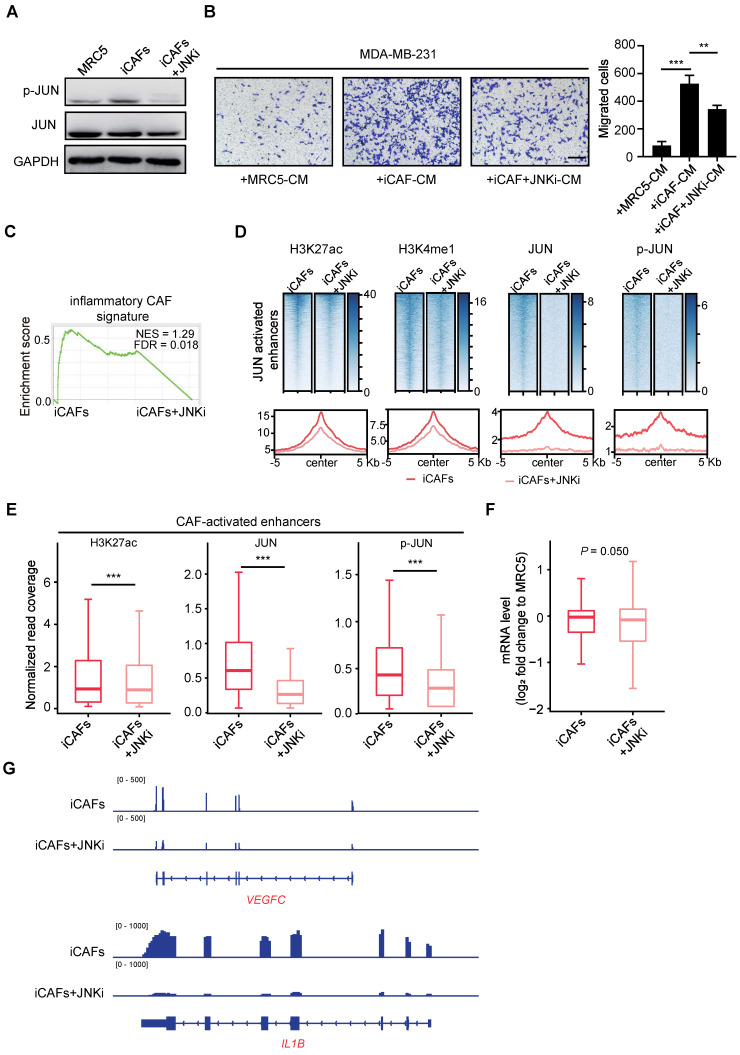
** Phosphorylated JUN is required for maintenance of the CAF-activated enhancers.** (**A**) WB assays showing the levels of p-JUN and JUN in mock MRC5 cells, induced CAFs (iCAFs) and JNKi-treated iCAFs (iCAFs+JNKi). GAPDH is used as a loading control. (**B**) Transwell assays showing migrated MDA-MB-231 after incubation with CM of MRC5, iCAFs and iCAFs+JNKi for 48 h. Left: representative images are shown. Scale bars, 200 μm. Right: quantification analysis of migrated cells is shown. Bars represent mean ± SD, *n* = 3. *P* value was determined by two-sided unpaired *t* test. **, *P* < 0.01; ***, *P* < 0.001. (**C**) GSEA for inflammatory CAF signature based on the RNA-seq data of iCAFs and iCAFs+JNKi. (**D**) Heatmaps and average profiles of H3K27ac, H3K4me1, JUN and p-JUN ChIP-seq signals across regions of ± 5,000 bp surrounding JUN-activated enhancers center. (**E**) Boxplots to compare the enrichment of H3K27ac, JUN and p-JUN at CAF-activated enhancers in each designated group of cells. *P* value was determined by two-sided paired *t* test. ***, *P* < 0.001. (**F**) The differential mRNA levels of JUN-activated enhancers nearest genes (log_2_ fold change (each group to MRC5 control cells)) is shown as boxplot. *P* value was determined by one-sided paired* t* test. (**G**) Tracks of RNA-seq signals of designated genes respectively in mock iCAFs and JNKi-treated iCAFs.

After showing that JUN-activated transcriptional network induces inflammatory CAF activation, we followed to find out whether JUN activity is required for maintenance of the CAF-activated enhancers and expression profiles. To do this, we first managed to establish an induced CAFs (iCAFs) model through culturing immortalized MRC5 cells with MDA-MB-231-derived CM for 7 days. As detected by WB assays, p-JUN levels were elevated in iCAFs compared with the uninduced MRC5 cells, whereas total JUN protein levels remained unchanged (Figure 5A). And this elevation of p-JUN levels could be abrogated by 48 h treatment of iCAFs with JNK-IN-8 (JNKi), a selective JNK inhibitor that inhibits phosphorylation of JUN 49. Moreover, transwell assays showed that the breast cancer cells incubated with iCAF-CM showed a much stronger migration-promoting ability than the one incubated with MRC5-CM, and this was significantly reversed by JNKi treatment (Figure 5B). Consistently, GSEA analysis showed that JNKi treatment in iCAFs specifically affected the enrichment of inflammatory CAF-related signature, but not myofibroblastic CAF signature (Figure 5C and Figure S8A). Thus, JUN activation is indispensable for the maintenance of inflammatory CAF-like features.

Given that these models offered us an option to reversibly manipulate JUN transcription activity, we followed to find out how inhibition of JUN activation would affect the maintenance of JUN-activated enhancers. Accordingly, we performed ChIP-seq analyses for enhancer marks and JUN chromatin binding in iCAFs treated with or without JNKi. After JNKi treatment, H3K27ac enrichment at JUN-activated enhancers was significantly decreased (Figure 5D and Figure S8B). Accumulation of H3K4me1 was also inhibited (Figure 5D). Correspondingly, JNKi led to strikingly reduced occupancy of JUN and p-JUN at these regions (Figure 5D and Figure S8B). Meanwhile, we also turned to analyze how JNKi treatment of iCAFs might affect the chromatin states at the CAF-activated enhancers. As shown in Figure 5E, a significant reduction of H3K27ac enrichment and JUN binding levels was observed in JNKi-treated cells. Moreover, independent ChIP-qPCR analysis confirmed that JNKi induced significantly decreased enrichment of H3K27ac, total JUN and p-JUN at the associated enhancers (Figure S8C). Therefore, JUN activity is required for its proper chromatin occupancy and maintenance of the CAF-specific enhancers.

Then we followed to quantify the effects of JUN inactivation on gene expression. Focusing on JUN-activated enhancers-associated genes, we compared their expression levels in iCAFs treated with or without JNKi. As shown in Figure 5F, JNKi significantly reduced the expression levels of JUN-activated enhancers-associated genes. For instance, the expression of two key downstream target genes of JUN, *VEGFC* and *IL1B* are strongly suppressed by JNKi treatment (Figure 5G). Together, these data indicate that inhibition of JUN activity diminishes the CAF molecular and functional features *in vitro*.

### JUN deficiency in stroma inhibits tumor metastasis *in vivo*

**Figure 6:**
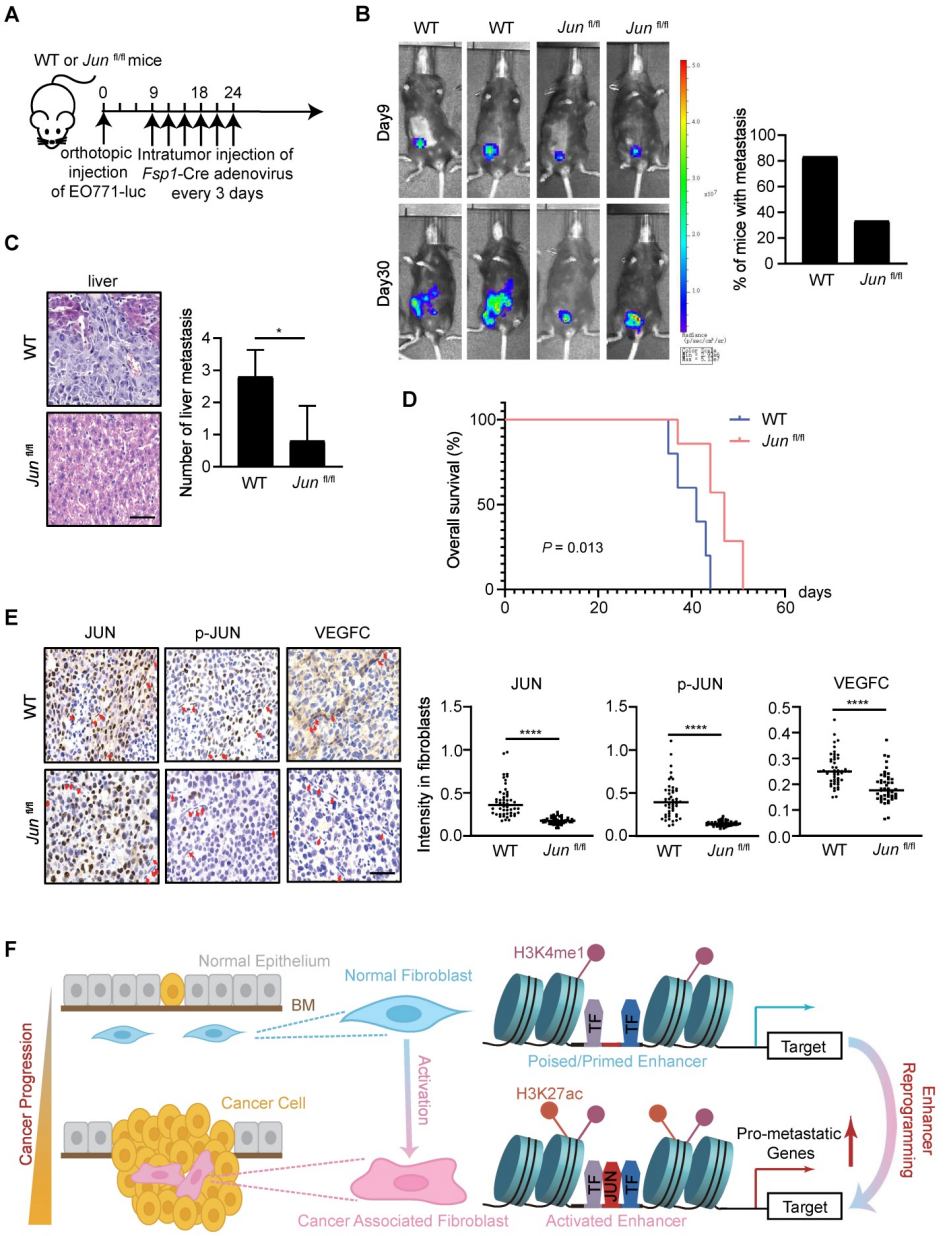
** JUN deficiency in stroma inhibits cancer metastasis *in vivo*.** (**A**) Schematics showing the experimental design. (**B**) Left: representative luciferase images of allografted mice, 9 and 30 days after tumor implantation. Right: quantification of the number of mice with metastasis, *n* = 6 per group. (**C**) Left: H&E staining of allografted tumors showing liver metastasis. Scale bars, 50 μm. Right: quantification of the number of liver metastasis. Bars represent mean ± SD.* P* value was calculated using two-sided unpaired *t* test. *, *P* < 0.05, *n* = 5 per group. (**D**) Kaplan-Meier survival curve of allografted mice. *P* value was determined by log-rank test, *n* = 6 per group. (**E**) Left: immunostaining of JUN, p-JUN, VEGFC in allografted tumors. Fibroblasts are labeled using red arrows. Scale bars, 50 μm. Right: quantification of immunostaining intensities in fibroblasts. Line represents the median. *P* value was calculated using two-sided unpaired *t* test. ****, *P* < 0.0001, *n* = 50. (**F**) A proposed model of JUN-mediated enhancer reprogramming which underlies the upregulated expression of pro-metastatic genes. Consequently, this deregulated transcription regulatory network results in CAF activation and thereby non-autonomously promoters breast cancer metastasis. BM, base membrane.

To further test the roles of stromal JUN activation in tumor progression, we tried to examine how fibroblast-specific loss of JUN affects breast cancer progression. In short, EO771, a tumor cell line derived from spontaneous breast cancer of C57BL/6 mice 50, 51, was stably transduced with luciferase-expressing lentivirus and injected into the mammary fat pad of WT and *Jun*^fl/fl^ mice to generate a breast cancer orthotopic model. At the onset of tumors (around 9 days after transplantation), intratumoral injection of adenovirus expressing fibroblasts-specific Cre adenovirus (*Fsp1*-Cre) was administrated every 3 days. Subsequently, tumor progression was monitored every week (Figure 6A and Figure S9).

As examined by luciferase activity and histological analysis of liver tissues, the tumor dissemination was significantly obliterated by stromal JUN deletion (Figure 6B-C). Accordingly, substantially prolonged survival of the transplanted mice was observed (Figure 6D). In line with transcription regulatory effects in cell models, IHC analysis of tumor samples showed a significant reduction of VEGFC protein levels in JUN-deficient stroma (Figure 6E). Therefore, JUN deficiency in stroma indeed inhibits tumor progression and metastasis *in vivo*. More careful analyses are required to find out whether and how other CAF signature genes are influenced in fibroblasts, and how cancer cell fates are reprogrammed in this context.

## Discussion

How stromal cells are recruited and/or reprogrammed in favor of tumor progression and therapeutic resistance is an important question in cancer biology. In this study, we take advantage of transcriptomic and epigenomic approaches to systemically unveil the common features of CAFs associated with breast cancer metastasis. And through* in vitro* and *in vivo* models, we demonstrate that enhancer reprogramming is accompanied with CAF activation during cancer progression. Specifically, we find that activated JUN in stroma is necessary and sufficient to remodel CAF-specific enhancer landscape, promotes the expression of pro-metastatic genes and thereby augments breast cancer invasiveness (Figure 6F).

So far, distinct subtypes of CAFs have been identified. Even their roles in cancer progression are controversial 47, 52-55. Thus, CAF heterogeneity is a non-negligible issue. Consistently, the transcriptomic dataset that we generated from paired NFs and CAFs did show inter-tumoral heterogeneity. Nevertheless, the dataset still provides valuable resources for the research community to get a glimpse into TME of breast cancers. Apparently, the common features that distinguish CAFs from NFs are of clinical significance (Figure 1 and Figure S1). This is reconcilable with the cellular heterogeneity when considering that subtypes of CAFs may exist in each tumor in varied ratios, though these CAFs do share certain invasive molecular features. Single cell analyses in defined cancer subtypes in the future may provide a clearer picture of CAF commonness and heterogeneity. And the values of the defined CAF-specific gene signature await to be tested in further broad studies.

In comparision with transcriptomic profiles, the epigenomic features have been rarely unveiled for CAFs, probably due to limited numbers of purified cells available. The development of epigenomic approaches with ultra-low inputs has made this feasible in recent years. For instances, distinct and enduring DNA methylation changes have ever been identified in CAFs isolated from prostate cancer samples 56. In addition, locus-specific loss of H3K27me3 were found at the promoters of genes encoding stem cell niche factors, cell growth, tissue development and stromal-epithelial interactions, etc, accompanied with their derepressed gene expression 57. However, these chromatin changes are more likely a consequence of CAF activation. Here considering of the crucial roles of enhancers in determining cell type or state specificity, we have carefully profiled enhancer marks in paired NFs and CAFs at the genome-wide for the first time. According to our data, the CAF-activated enhancers that clearly separate CAFs from NFs are defined. Notably, the CAF-activated enhancers are reconfigured from pre-existing primed or poised enhancers with pre-occupied lineage-defining TFs, as there lack apparent differences in H3K4me1 enrichment between NFs and CAFs (Figure 6F). It supports a previously raised concept that CAF should be regarded as an active state, rather than a cell type 58.

Based on the defined CAF-activated enhancers, we identify AP-1 as the key driving factors. Though the functions and transcription regulatory mechanisms for AP-1 are well documented in cancer cells 36, its roles in stroma have remained poorly characterized. In response to the education by cancer cells, signal-regulated TFs like JUN are likely to be activated in CAFs. Whereas in NFs, the inactive JUN may interact with Mbd3/NuRD repressor complex and remain unfunctional 59. In support, two recent studies have illustrated that activated JUN contributes to selection and establishment of specific enhancers in fibroblasts 37, 60, which is consistent with our findings. And through genetic and chemical approaches, we demonstrate that JUN phosphorylation at the transactivation domain is indispensable for the establishment and maintenance of CAF-activated enhancers. Accordingly, specific inhibition of JUN in fibroblasts successfully suppresses CAF functions, for example, VEGFC expression and secretion, and thereby curbs cancer development. It would be exciting to further carefully examine whether and how JNKi administration in allografted tumor models would affect TME, and to measure the efficacies of potential combinations with other radiochemical therapies.

To summarize, based on a transcriptomic and epigenomic view of CAFs associated with invasive breast cancers, we have unveiled the significance of AP-1 centered transcription regulatory network in CAF activation and cancer progression. Therefore, targeting AP-1 may provide an anti-stroma as well as anti-cancer therapeutic strategy.

## Methods

### Patients, tissue samples and primary cell culture

Tumor tissues and corresponding para-tumoral non-malignant tissues were surgically removed from invasive breast cancer patients with axillary lymph node metastasis at Union Hospital, Wuhan. None of the patients have received radiotherapy or chemotherapy before surgery. The clinical features of patients were provided in Table S1.

Primary CAFs and NFs were isolated as previously described 52, 61. Briefly, tissues were cut into small pieces and digested using collagenase type I/III and hyaluronidase (1 mg/mL, Sigma Aldrich) for 2-3 h at 37 °C. Cells were collected by centrifugation at 250 g for 5 min, then resuspended in the growth medium. After an hour of culture, stroma cells-enriched supernatant was removed to a new tube. Human fibroblasts were obtained by centrifuge at 250 g for 5 min and then cultured in the growth medium. The purity of isolated fibroblasts was confirmed by IF staining. Primary NFs and CAFs were positive for vimentin (> 95%). Second- to fifth-passage of primary fibroblasts were employed in our study.

### Cell culture and conditional medium preparation

Human embryonic lung fibroblasts MRC5 (American Type Culture Collection, ATCC) cells were immortalized by transduction with hTERT-expressing lentivirus (Shanghai GeneChem Co., Ltd, Shanghai, China). EO771, a tumor cell line which is derived from spontaneous breast cancer of C57BL/6 mice, was kindly provided by Prof. Yuhui Huang from Soochow University, Suzhou, China. Human breast cancer cell line MDA-MB-231 (ATCC), immortalized MRC5, primary fibroblasts and EO771 were maintained in DMEM medium containing 10% FBS in a humidified 5% CO2 environment at 37 °C. To generate JUN WT or JUN AA overexpressed fibroblasts, The control, FLAG-tagged JUN WT or AA overexpression adenovirus were prepared by Hanbio biotechnology Company (Shanghai, China). MRC5 cells were infected with adenovirus expressing mock control or FLAG-tagged JUN WT or AA for 2 h at 37 °C and then cultured for 3 days.

About 70%-80% confluent cancer cells or fibroblasts were incubated with DMEM medium without FBS. One day later, conditional medium (CM) was harvested and filtered. Induced CAFs (iCAFs) were generated by culturing MRC5 cells in MDA-MB-231-derived CM for 7 days. To inhibit JUN phosphorylation, iCAFs were treated with JNKi (1 µM, MedChemExpress) for 48 h. If used for CM preparation, the cells were washed twice with culture medium to remove residual JNKi.

### Immunofluorescence

Cells were fixed with 4% formaldehyde and permeabilized with PBS containing 0.1% Triton X-100. Then it was blocked in PBS containing 5 mg/mL BSA at room temperature (RT) and incubated with primary antibodies overnight at 4 °C. Primary antibodies used in our study are as below. Vimentin antibody (1:200, Cell Signaling Technology, catalog 5741); ɑ-SMA (1:500, Sigma Aldrich, catalog A2547); c-Jun antibody (1:200, Cell Signaling Technology, catalog 9165S); c-Jun (phospho S63) antibody (1:200, Abcam, catalog ab32385). After incubation with FITC or TRITC-secondary antibody for 1 h at RT, the cells were counterstained by DAPI and images were obtained.

### Western blot

Protein lysate was resolved by SDS-polyacrylamide gels, followed by membranes transfer. Primary antibodies used in our study included c-Jun antibody (1:1,000, Cell Signaling Technology, catalog 9165S), c-Jun (phospho S63) antibody (1:1,000, Abcam, catalog ab32385) and GAPDH antibody (1:20,000, Abclonal, catalog AC002). Followed by peroxidase conjugated secondary antibody incubation, it was visualized by enhanced chemiluminescence assay.

### Transwell assay

Transwell chambers with 8-µm pore size in 24-well plates (Corning) were used to perform migration assay as described 62. The chambers pre-coated with matrigel were used to perform invasion assay. After incubation with appropriate CM for 48 h, 5×10^4^ MDA-MB-231 cells were seeded in the upper chamber with serum-free medium. The lower chamber was filled with 600 µl DMEM with 5% FBS as a chemoattractant. After 24 h of incubation in the incubator, cells in the upper chamber were removed carefully with a cotton swab. The migrated and invaded cells were washed twice with PBS, fixed and stained with 0.1% crystal violet for 10 min, followed by image capture and quantification.

### Human tissue array, histology and quantitative analysis

Human tissue array containing pairs of invasive breast carcinoma tissues and matched para-tumoral non-malignant tissues was purchased from Shanghai WellBio technology Co., Ltd, Shanghai, China. Tissue sections were deparaffinized and rehydrated. The slides were stained with hematoxylin and eosin. For immunohistochemistry (IHC), the slides were incubated with primary antibodies overnight at 4 °C, followed antigen retrieval and blocking. Primary antibodies used in our study are as below. c-Jun antibody (1:200, Cell Signaling Technology, catalog 9165S), phospho-c-Jun (Ser73) antibody (1:200, Cell Signaling Technology, catalog 3270S), VEGFC antibody (1:200, Abclonal, catalog A2556), FSP1 antibody (1:500, Cell Signaling Technology, catalog 13018S). The staining was visualized by HRP-conjugated secondary antibody and DAB substrate kit (ZSGB-BIO, Beijing, China). Quantification of IHC staining was determined using Image J Fiji software.

IHC staining was scored considering both percentage of positive stained cells and staining intensity. Each section was scored independently without prior knowledge of patient information. Stroma JUN and p-JUN score was determined by multiplying the score for percentage of positive cells in stroma with the score for staining intensity in stroma cells. The detailed criterion of scoring for percentage of positive cells: (i) 0%-25%; (ii) 26%-50%; (iii) 51%-75%; (iv) 76%-100% of the stroma cells showed positive staining. The detailed criterion of scoring for staining intensity: (i) negative; (ii) weak; (iii) moderate; (iv) strong.

### Cleavage Under Targets and Tagmentation (CUT&Tag)

CUT&Tag assay was carried out as described previously 33, 63. In brief, 30,000 primary fibroblasts were harvested. The cells were washed and resuspended in 300 µl wash buffer. Concanavalin A coated beads were activated through washing in binding buffer. The cells were incubated with 10 µl activated beads for 30 min at RT. After discarding the supernatant, beads were incubated with 0.5 µl primary antibody in 50 µl antibody buffer for 2 h at RT. After discarding the supernatant gently, beads were resuspended and incubated with 1 µl Guinea Pig anti-Rabbit IgG antibody in 100 µl Dig wash buffer for 1 h at RT. Followed by washing the beads using Dig wash buffer, 100 µl Dig 300 wash buffer was used to resuspend the beads. After incubation with 0.04 μM pA-Tn5 adapter complex (Vazyme S603) for 1 h at RT, Dig 300 wash buffer was used to wash the beads three times. Then the beads were incubated with 300 µl tagmentation buffer for 1 h at 37 °C. After tagmentation was stopped, DNA was extracted and amplified with i5 and i7 primer in KAPA 2×PCR mix (KM2602). DNA was size-selected to enrich 200-1,000 bp fragments by Ampure XP beads. The purified DNA was used to construct libraries for sequencing. H3K4me1 (Cell Signaling Technology, catalog 5326P) and H3K27ac (Abcam, catalog ab4729) antibodies were used in our CUT&Tag assay.

### Chromatin immunoprecipitation (ChIP)-qPCR

Chromatin preparation was performed as described previously 64. Briefly, cells were cross-linked with 1% formaldehyde for 10 min at RT. For selected experiments (ChIP for JUN and p-JUN), cells were sequentially crosslinked with 2 mM EGS (Ethylene glycol-bis (succinic acid N-hydroxysuccinimide ester), Sigma Aldrich, catalog E3257) and 1% formaldehyde for 15 min at RT. Then it was quenched by 0.125 M glycine for 5 min. After being washed twice by PBS, the cell pellets were resuspended, lysed and chromatin was sheared with BioRuptor sonicator (Diagenode). Primary antibodies were added to the pre-cleared chromatin overnight at 4 °C. H3K4me1, c-Jun and phospho-c-Jun (Ser73) antibody were purchased from Cell Signaling Technology (catalog 5326P, 9165S, 3270S). H3K27ac antibody was purchased from Abcam (catalog ab4729). Then 30 µl protein A/G magnetic beads (Bimake, catalog B23202) were added for IP. After 2 h incubation at 4 °C, the beads were washed three times by washing buffer. ChIP DNA was purified using PCR purification kits (QIAGEN, Hilden, Germany) after reverse cross-linking at 65 °C overnight. Primers for qPCR analysis are as below. For associated enhancers of *IL1B*: forward, 5'-AACCGTAGTATCGCACCCAC-3'; reverse, 5'-TCCCCAGCACAGGAAGTTTG-3'. For associated enhancers of *VEGFC*: forward, 5'-ATCAAGGACTCAAATTATCA-3'; reverse, 5'-AGAACAGACTGCATTCTGTG-3'. Data were obtained and analyzed by Roche LightCycler 480 instrument.

### RNA-seq and data analysis

Total RNA was extracted using TRIzol (Invitrogen). Library construction and sequencing were carried out by Beijing Genomics Institute (BGI). Reads were mapped to the reference human genome assembly hg19 using hisat2 version 2.1.0 with default parameters 65. Assembling full-length transcripts and quantifications for each gene to generate Fragments Per Kilobase of exon model per Million mapped fragments (FPKM) values were performed by StringTie v2.1.1 66. For GSVA analyses of RNA-seq data from 7 pairs of primary NFs and matched CAFs, FPKM values were log_2_-transformed. The analyses were carried out by R package gsva (method = 'ssgsea') against MSigDB gene sets v7.3 28. Log_2_-transformed fold change (CAFs/paired NFs) of each patient was calculated to generate heatmaps. The genes whose fold change (paired CAFs/NFs) in mRNA expression levels ≥ 2 in > 3 pairs were defined as CAF signature genes. The primary fibroblasts were unsupervised hierarchical clustered by mRNA expression levels of CAF signature genes.

### GSEA analysis

GSEA analysis was performed using gene sets from MSigDB gene sets v7.3 and literature 48 with default settings 45, 46.

### Microarray data and TCGA data analysis

Processed expression values of microarray data for normal stroma and invasive stroma were retrieved from GEO website (GSE14548). CAF signature score was calculated as mean of CAF signature genes' mRNA levels, with removing undetected genes in microarray. Transcriptome data for The Cancer Genome Atlas (TCGA) were obtained for survival analyses. R package survival was used to determine the optimal cutpoint and compare the overall survival rate between patients with higher and lower expressed gene. *P* value was determined using Log-rank test.

### ChIP-seq data analysis

Raw reads were aligned to the reference human genome assembly hg19 using Bowtie2 with default parameters 67. Only unique mapped reads with mapping quality greater than 20 were subjected to further analysis. Samtools was used to remove duplicated mapped reads 68. Differential H3K27ac peaks between JUN WT overexpression group and control were obtained through MACS2 bdgdiff 69 with '--cutoff 8' parameter and considered as JUN-activated enhancers. Quantitation of enrichment of H3K27ac, H3K4me1, JUN and p-JUN was determined by normalization of the mapped reads at appropriate intervals to one million of total unique reads.

### CUT&Tag sequencing analysis

Raw reads were mapped to the reference human genome assembly hg19 by Bowtie2 with '--local --very-sensitive --no-mixed --no-discordant -I 10 -X 700' parameters 67. Only unique mapped reads with mapping quality greater than 20 were subjected to following analysis. Samtools was used to remove duplicated mapped reads 68. Peak calling was performed by MACS2 callpeak 69.

For calculating changed H3K27ac peaks in each patient, mergePeaks tool in HOMER suite (maximum distance between peak centers to merge: 200 bp) was used to combine NFs and matched CAFs H3K27ac peaks of each patient. H3K27ac enrichment was calculated by normalization of the mapped reads at the interval to one million of total unique reads. Peaks with increased and decreased H3K27ac enrichment in each patient was defined with fold change (CAFs/paired NFs) ≥ 2 and fold change (CAFs/paired NFs) ≤ 1/2, respectively. Peaks annotation was performed by R package ChIPseeker 70, with defining Transcription Start Site (TSS) ± 1Kb as promoter regions.

For calculating variable H3K27ac enrichment in all patients, mergePeaks tool (maximum distance between peak centers to merge: 200 bp) was used to combine H3K27ac peaks in all of primary fibroblasts. Promoter regions and non-promoter regions were identified by ChIPseeker 70, with defining TSS ± 1Kb as promoter regions. Fold change (CAFs/paired NFs) of H3K27ac enrichment in each patient was calculated. After ranking the sum of fold change in all patients, TOP 2,000 regions were identified. PCA was performed by H3K27ac enrichment of TOP 2,000 promoter regions and TOP 2,000 non-promoter regions in R, respectively.

For analyzing active enhancers in primary fibroblasts, H3K27ac peaks which overlapped with H3K4me1 peaks at least 1 bp in each sample were regarded as active enhancers in this sample. The mergePeaks tool (maximum distance between peak centers to merge: 200 bp) was used to combine active enhancers in all of primary fibroblasts. The union of active enhancers (All enhancers, *n* = 87,108) were yielded. H3K27ac enrichment in each sample was calculated by normalization of the mapped reads at All enhancers to one million of total unique reads. Filtered by fold change of H3K27ac enrichment (CAFs/paired NFs) ≥ 2 in > 3 pairs, CAF-activated enhancers (*n* = 3,333) were identified. CAF-repressed enhancers (*n* = 1,881) were determined according to fold change of H3K27ac enrichment (CAFs/paired NFs) ≤ 1/2 in > 3 pairs. PCA analysis and unsupervised hierarchical clustering of all primary fibroblasts were performed by H3K27ac enrichment at CAF-activated enhancers.

### Displaying CUT&Tag sequencing and ChIP-seq data

For CUT&Tag sequencing and ChIP-seq data, heatmaps and average profiles of H3K27ac, H3K4me1, JUN and p-JUN were generated by deeptools v3.5.1 71.

### Associated genes and Kyoto Encyclopedia of Genes and Genomes (KEGG) enrichment analysis

Genes associated with identified regions were determined using their nearest gene. KEGG enrichment analysis was carried out by R package clusterProfiler 72.

### Motif analysis of CAF-activated enhancers

For motif discovery, genomic sequences of ± 300 bp surrounding CAF-activated enhancers center was extracted from the hg19 genome. These sequences were analyzed to identify enriched motifs by CentriMo 34 of MEME suite, with JASPAR CORE vertebrates motifs. The findMotifs.pl program in HOMER was also employed to motif analysis in these sequences, with default parameters 35.

### Sequencing data visualization

For visualization of sequencing data, processed bam file for each sample was transformed to a bigwig file by deeptools bamCoverage, with Reads Per Kilobase per Million mapped reads (RPKM) normalization. For visualization of RNA-seq and CUT&Tag sequencing data in primary fibroblasts, paired NFs-subtracted bigwig files were generated by deeptools bigwigCompare (-b1 'bigwig file of CAFs' -b2 'bigwig file of paired NFs' --operation subtract ) 71. All data were further visualized using Integrative Genomics Viewer (IGV).

### Generation of *Fsp1*-Cre adenovirus

A portion of the *Fsp1* genome containing 2,500 bp of *Fsp1* 5' flanking region and the first intron, as well as the noncoding portions of the first and second exon 73 was cloned upstream of Cre recombinase coding sequence in adenovirus shuttle vector. Plasmid construction and adenovirus packaging were provided by Hanbio BioTechnology Company, Shanghai, China.

### *In vivo* xenograft experiments

*Jun*^fl/fl^ mice were kindly provided by Prof. Feifan Guo from Shanghai Institute of Nutrition and Health, Chinese Academy of Sciences. Six- to eight-week-old female C57BL/6 genetic background-wildtype or -*Jun*^fl/fl^ mice were housed in animal facility at Tianjin Medical University. EO771 cells were infected with lentivirus expressing Luciferase to generate EO771-luc cells. A total of 6×10^4^ EO771-luc cells resuspended in 100 μl of 1:1 mix of PBS and matrigel were injected into mammary fat pad of WT mice or *Jun*^fl/fl^ mice to generate breast cancer orthotopic xenografts. When the tumors were just visible at day 9, intratumor injection of *Fsp1*-Cre adenovirus was administrated every 3 days. A total of 6 times of administration were performed. Tumors were monitored by Xenogen bioluminescence imaging every week. Xenograft tumors were embedded in paraffin and used for IHC staining.

### Data availability

All deep sequencing raw data of this study have been deposited in the Gene Expression Omnibus (GEO) under accession code GSE196404.

### Statistics

The detailed information about statistical analysis is indicated in the figure legends. Statistical values were determined in R or Graphpad Prism 8. Error bars in the experiments indicate standard deviation (SD) of the mean for three independent experiments. *P* value less than 0.05 was considered statistically significant.

### Study approval

Before the isolation of samples from breast cancer patients, informed consent was obtained in accordance with the Declaration of Helsinki and the protocol was approved by the Institutional Review Board of Wuhan Union hospital. All animal studies were performed according to Health guidelines of Tianjin Medical University Institutional Animal Use and Care Committee.

## Supplementary Material

Supplementary figures and tables.

## References

[1] Bejarano L, Jordao MJC, Joyce JA (2021). Therapeutic targeting of the tumor microenvironment. Cancer Discov.

[2] Hinshaw DC, Shevde LA (2019). The tumor microenvironment innately modulates cancer progression. Cancer Res.

[3] Calon A, Lonardo E, Berenguer-Llergo A, Espinet E, Hernando-Momblona X, Iglesias M (2015). Stromal gene expression defines poor-prognosis subtypes in colorectal cancer. Nat Genet.

[4] Isella C, Terrasi A, Bellomo SE, Petti C, Galatola G, Muratore A (2015). Stromal contribution to the colorectal cancer transcriptome. Nat Genet.

[5] Roswall P, Bocci M, Bartoschek M, Li H, Kristiansen G, Jansson S (2018). Microenvironmental control of breast cancer subtype elicited through paracrine platelet-derived growth factor-CC signaling. Nat Med.

[6] Su S, Liu Q, Chen J, Chen J, Chen F, He C (2014). A positive feedback loop between mesenchymal-like cancer cells and macrophages is essential to breast cancer metastasis. Cancer Cell.

[7] Scherz-Shouval R, Santagata S, Mendillo ML, Sholl LM, Ben-Aharon I, Beck AH (2014). The reprogramming of tumor stroma by HSF1 is a potent enabler of malignancy. Cell.

[8] Chen X, Song E (2019). Turning foes to friends: targeting cancer-associated fibroblasts. Nat Rev Drug Discov.

[9] Loibl S, Poortmans P, Morrow M, Denkert C, Curigliano G (2021). Breast cancer. Lancet.

[10] Sahai E, Astsaturov I, Cukierman E, DeNardo DG, Egeblad M, Evans RM (2020). A framework for advancing our understanding of cancer-associated fibroblasts. Nat Rev Cancer.

[11] Chen Y, McAndrews KM, Kalluri R (2021). Clinical and therapeutic relevance of cancer-associated fibroblasts. Nat Rev Clin Oncol.

[12] Kalluri R (2016). The biology and function of fibroblasts in cancer. Nat Rev Cancer.

[13] Erez N, Truitt M, Olson P, Arron ST, Hanahan D (2010). Cancer-associated fibroblasts are activated in incipient neoplasia to orchestrate tumor-promoting inflammation in an NF-kappaB-dependent manner. Cancer Cell.

[14] Cazet AS, Hui MN, Elsworth BL, Wu SZ, Roden D, Chan CL (2018). Targeting stromal remodeling and cancer stem cell plasticity overcomes chemoresistance in triple negative breast cancer. Nat Commun.

[15] Arina A, Idel C, Hyjek EM, Alegre ML, Wang Y, Bindokas VP (2016). Tumor-associated fibroblasts predominantly come from local and not circulating precursors. Proc Natl Acad Sci U S A.

[16] Calon A, Espinet E, Palomo-Ponce S, Tauriello DV, Iglesias M, Cespedes MV (2012). Dependency of colorectal cancer on a TGF-beta-driven program in stromal cells for metastasis initiation. Cancer Cell.

[17] Biffi G, Oni TE, Spielman B, Hao Y, Elyada E, Park Y (2019). IL1-induced JAK/STAT signaling is antagonized by TGFbeta to shape CAF heterogeneity in pancreatic ductal adenocarcinoma. Cancer Discov.

[18] Karakasheva TA, Lin EW, Tang Q, Qiao E, Waldron TJ, Soni M (2018). IL-6 mediates cross-talk between tumor cells and activated fibroblasts in the tumor microenvironment. Cancer Res.

[19] Sharon Y, Raz Y, Cohen N, Ben-Shmuel A, Schwartz H, Geiger T (2015). Tumor-derived osteopontin reprograms normal mammary fibroblasts to promote inflammation and tumor growth in breast cancer. Cancer Res.

[20] Valenti G, Quinn HM, Heynen G, Lan L, Holland JD, Vogel R (2017). Cancer stem cells regulate cancer-associated fibroblasts via activation of hedgehog signaling in mammary gland tumors. Cancer Res.

[21] Min J, Zaslavsky A, Fedele G, McLaughlin SK, Reczek EE, De Raedt T (2010). An oncogene-tumor suppressor cascade drives metastatic prostate cancer by coordinately activating Ras and nuclear factor-kappaB. Nat Med.

[22] Ernst J, Kheradpour P, Mikkelsen TS, Shoresh N, Ward LD, Epstein CB (2011). Mapping and analysis of chromatin state dynamics in nine human cell types. Nature.

[23] Heintzman ND, Hon GC, Hawkins RD, Kheradpour P, Stark A, Harp LF (2009). Histone modifications at human enhancers reflect global cell-type-specific gene expression. Nature.

[24] Buecker C, Wysocka J (2012). Enhancers as information integration hubs in development: lessons from genomics. Trends Genet.

[25] Shlyueva D, Stampfel G, Stark A (2014). Transcriptional enhancers: from properties to genome-wide predictions. Nat Rev Genet.

[26] Roe JS, Hwang CI, Somerville TDD, Milazzo JP, Lee EJ, Da Silva B (2017). Enhancer reprogramming promotes pancreatic cancer metastasis. Cell.

[27] Liu F, Hon GC, Villa GR, Turner KM, Ikegami S, Yang H (2015). EGFR mutation promotes glioblastoma through epigenome and transcription factor network remodeling. Mol Cell.

[28] Hänzelmann S, Castelo R, Guinney J (2013). GSVA: gene set variation analysis for microarray and RNA-seq data. BMC Bioinformatics.

[29] Korkaya H, Liu S, Wicha MS (2011). Breast cancer stem cells, cytokine networks, and the tumor microenvironment. J Clin Invest.

[30] Wu Y-H, Huang Y-F, Chang T-H, Chen C-C, Wu P-Y, Huang S-C (2021). COL11A1 activates cancer-associated fibroblasts by modulating TGF-β3 through the NF-κB/IGFBP2 axis in ovarian cancer cells. Oncogene.

[31] Zhang T, Li X, He Y, Wang Y, Shen J, Wang S (2022). Cancer-associated fibroblasts-derived HAPLN1 promotes tumour invasion through extracellular matrix remodeling in gastric cancer. Gastric Cancer.

[32] Zhu HF, Zhang XH, Gu CS, Zhong Y, Long T, Ma YD (2019). Cancer-associated fibroblasts promote colorectal cancer progression by secreting CLEC3B. Cancer Biol Ther.

[33] Kaya-Okur HS, Wu SJ, Codomo CA, Pledger ES, Bryson TD, Henikoff JG (2019). CUT&Tag for efficient epigenomic profiling of small samples and single cells. Nat Commun.

[34] Bailey TL, Machanick P (2012). Inferring direct DNA binding from ChIP-seq. Nucleic Acids Res.

[35] Heinz S, Benner C, Spann N, Bertolino E, Lin YC, Laslo P (2010). Simple combinations of lineage-determining transcription factors prime cis-regulatory elements required for macrophage and B cell identities. Mol Cell.

[36] Eferl R, Wagner EF (2003). AP-1: a double-edged sword in tumorigenesis. Nat Rev Cancer.

[37] Vierbuchen T, Ling E, Cowley CJ, Couch CH, Wang X, Harmin DA (2017). AP-1 transcription factors and the BAF complex mediate signal-dependent enhancer selection. Mol Cell.

[38] Madrigal P, Alasoo K (2018). AP-1 takes centre stage in enhancer chromatin dynamics. Trends Cell Biol.

[39] Zanconato F, Forcato M, Battilana G, Azzolin L, Quaranta E, Bodega B (2015). Genome-wide association between YAP/TAZ/TEAD and AP-1 at enhancers drives oncogenic growth. Nat Cell Biol.

[40] Bi M, Zhang Z, Jiang YZ, Xue P, Wang H, Lai Z (2020). Enhancer reprogramming driven by high-order assemblies of transcription factors promotes phenotypic plasticity and breast cancer endocrine resistance. Nat Cell Biol.

[41] Davies CC, Chakraborty A, Cipriani F, Haigh K, Haigh JJ, Behrens A (2010). Identification of a co-activator that links growth factor signalling to c-Jun/AP-1 activation. Nat Cell Biol.

[42] Markov GJ, Mai T, Nair S, Shcherbina A, Wang YX, Burns DM (2021). AP-1 is a temporally regulated dual gatekeeper of reprogramming to pluripotency. Proc Natl Acad Sci U S A.

[43] Angel P, Allegretto EA, Okino ST, Hattori K, Boyle WJ, Hunter T (1988). Oncogene jun encodes a sequence-specific trans-activator similar to AP-1. Nature.

[44] Smeal T, Binetruy B, Mercola DA, Birrer M, Karin M (1991). Oncogenic and transcriptional cooperation with Ha-Ras requires phosphorylation of c-Jun on serines 63 and 73. Nature.

[45] Subramanian A, Tamayo P, Mootha VK, Mukherjee S, Ebert BL, Gillette MA (2005). Gene set enrichment analysis: A knowledge-based approach for interpreting genome-wide expression profiles. Proc Natl Acad Sci U S A.

[46] Mootha VK, Lindgren CM, Eriksson K-F, Subramanian A, Sihag S, Lehar J (2003). PGC-1α-responsive genes involved in oxidative phosphorylation are coordinately downregulated in human diabetes. Nat Genet.

[47] Öhlund D, Handly-Santana A, Biffi G, Elyada E, Almeida AS, Ponz-Sarvise M (2017). Distinct populations of inflammatory fibroblasts and myofibroblasts in pancreatic cancer. J Exp Med.

[48] Elyada E, Bolisetty M, Laise P, Flynn WF, Courtois ET, Burkhart RA (2019). Cross-species single-cell analysis of pancreatic ductal adenocarcinoma reveals antigen-presenting cancer-associated fibroblasts. Cancer Discov.

[49] Zhang T, Inesta-Vaquera F, Niepel M, Zhang J, Ficarro SB, Machleidt T (2012). Discovery of potent and selective covalent inhibitors of JNK. Chem Biol.

[50] Sugiura K, Stock CC (1952). Studies in a tumor spectrum. I. Comparison of the action of methylbis (2-chloroethyl)amine and 3-bis(2-chloroethyl)aminomethyl-4-methoxymethyl -5-hydroxy-6-methylpyridine on the growth of a variety of mouse and rat tumors. Cancer.

[51] Le Naour A, Rossary A, Vasson MP (2020). EO771, is it a well-characterized cell line for mouse mammary cancer model? Limit and uncertainty. Cancer Med.

[52] Su S, Chen J, Yao H, Liu J, Yu S, Lao L (2018). CD10(+)GPR77(+) cancer-associated fibroblasts promote cancer formation and chemoresistance by sustaining cancer stemness. Cell.

[53] Costa A, Kieffer Y, Scholer-Dahirel A, Pelon F, Bourachot B, Cardon M (2018). Fibroblast heterogeneity and immunosuppressive environment in human breast cancer. Cancer Cell.

[54] Ozdemir BC, Pentcheva-Hoang T, Carstens JL, Zheng X, Wu CC, Simpson TR (2014). Depletion of carcinoma-associated fibroblasts and fibrosis induces immunosuppression and accelerates pancreas cancer with reduced survival. Cancer Cell.

[55] Rhim AD, Oberstein PE, Thomas DH, Mirek ET, Palermo CF, Sastra SA (2014). Stromal elements act to restrain, rather than support, pancreatic ductal adenocarcinoma. Cancer Cell.

[56] Pidsley R, Lawrence MG, Zotenko E, Niranjan B, Statham A, Song J (2018). Enduring epigenetic landmarks define the cancer microenvironment. Genome Res.

[57] Maeda M, Takeshima H, Iida N, Hattori N, Yamashita S, Moro H (2020). Cancer cell niche factors secreted from cancer-associated fibroblast by loss of H3K27me3. Gut.

[58] Madar S, Goldstein I, Rotter V (2013). 'Cancer associated fibroblasts'-more than meets the eye. Trends Mol Med.

[59] Aguilera C, Nakagawa K, Sancho R, Chakraborty A, Hendrich B, Behrens A (2011). c-Jun N-terminal phosphorylation antagonises recruitment of the Mbd3/NuRD repressor complex. Nature.

[60] Cui L, Chen SY, Lerbs T, Lee JW, Domizi P, Gordon S (2020). Activation of JUN in fibroblasts promotes pro-fibrotic programme and modulates protective immunity. Nat Commun.

[61] Orimo A, Gupta PB, Sgroi DC, Arenzana-Seisdedos F, Delaunay T, Naeem R (2005). Stromal fibroblasts present in invasive human breast carcinomas promote tumor growth and angiogenesis through elevated SDF-1/CXCL12 secretion. Cell.

[62] Dong F, Qin X, Wang B, Li Q, Hu J, Cheng X (2021). ALKBH5 facilitates hypoxia-induced paraspeckle assembly and IL8 secretion to generate an immunosuppressive tumor microenvironment. Cancer Res.

[63] Huo D, Yu Z, Li R, Gong M, Sidoli S, Lu X (2022). CpG island reconfiguration for the establishment and synchronization of polycomb functions upon exit from naive pluripotency. Mol Cell.

[64] Dong F, Li Q, Yang C, Huo D, Wang X, Ai C (2018). PRMT2 links histone H3R8 asymmetric dimethylation to oncogenic activation and tumorigenesis of glioblastoma. Nat Commun.

[65] Kim D, Paggi JM, Park C, Bennett C, Salzberg SL (2019). Graph-based genome alignment and genotyping with HISAT2 and HISAT-genotype. Nat Biotechnol.

[66] Pertea M, Pertea GM, Antonescu CM, Chang TC, Mendell JT, Salzberg SL (2015). StringTie enables improved reconstruction of a transcriptome from RNA-seq reads. Nat Biotechnol.

[67] Langmead B, Salzberg SL (2012). Fast gapped-read alignment with Bowtie 2. Nat Methods.

[68] Li H, Handsaker B, Wysoker A, Fennell T, Ruan J, Homer N (2009). The sequence alignment/map format and SAMtools. Bioinformatics.

[69] Zhang Y, Liu T, Meyer CA, Eeckhoute J, Johnson DS, Bernstein BE (2008). Model-based analysis of ChIP-Seq (MACS). Genome Biol.

[70] Yu G, Wang LG, He QY (2015). ChIPseeker: an R/Bioconductor package for ChIP peak annotation, comparison and visualization. Bioinformatics.

[71] Ramírez F, Ryan DP, Grüning B, Bhardwaj V, Kilpert F, Richter AS (2016). deepTools2: a next generation web server for deep-sequencing data analysis. Nucleic Acids Res.

[72] Yu G, Wang LG, Han Y, He QY (2012). clusterProfiler: an R package for comparing biological themes among gene clusters. OMICS.

[73] Okada H, Danoff TM, Fischer A, Lopez-Guisa JM, Strutz F, Neilson EG (1998). Identification of a novel cis-acting element for fibroblast-specific transcription of the FSP1 gene. Am J Physiol.

