# Supplementary material for: Enhancer reprogramming promotes the activation of cancer-associated fibroblasts and breast cancer metastasis
Source: Theranostics. 2022 Oct 24;12(17):7491–508. doi: 10.7150/thno.75853 (PMC9691365; doi:10.7150/thno.75853)
Supplement: Supplementary file 1 — Supplementary figures and tables. [file thnov12p7491s1.pdf]

Supplemental Material

Enhancer reprogramming promotes the activation of cancer-associated fibroblasts and breast cancer metastasis

Qian Li, Xuejiao Lv, Chunyong Han, Yu Kong, Zhongye Dai, Dawei Huo, Ting Li, Dapeng Li, Wei Li, Xing Wang, Qian Zhao, Jie Ming, Wen Yang, Yang Chen, Xudong Wu*

This file includes:

Figures S1-S9

Tables S1- S2

Figure S1

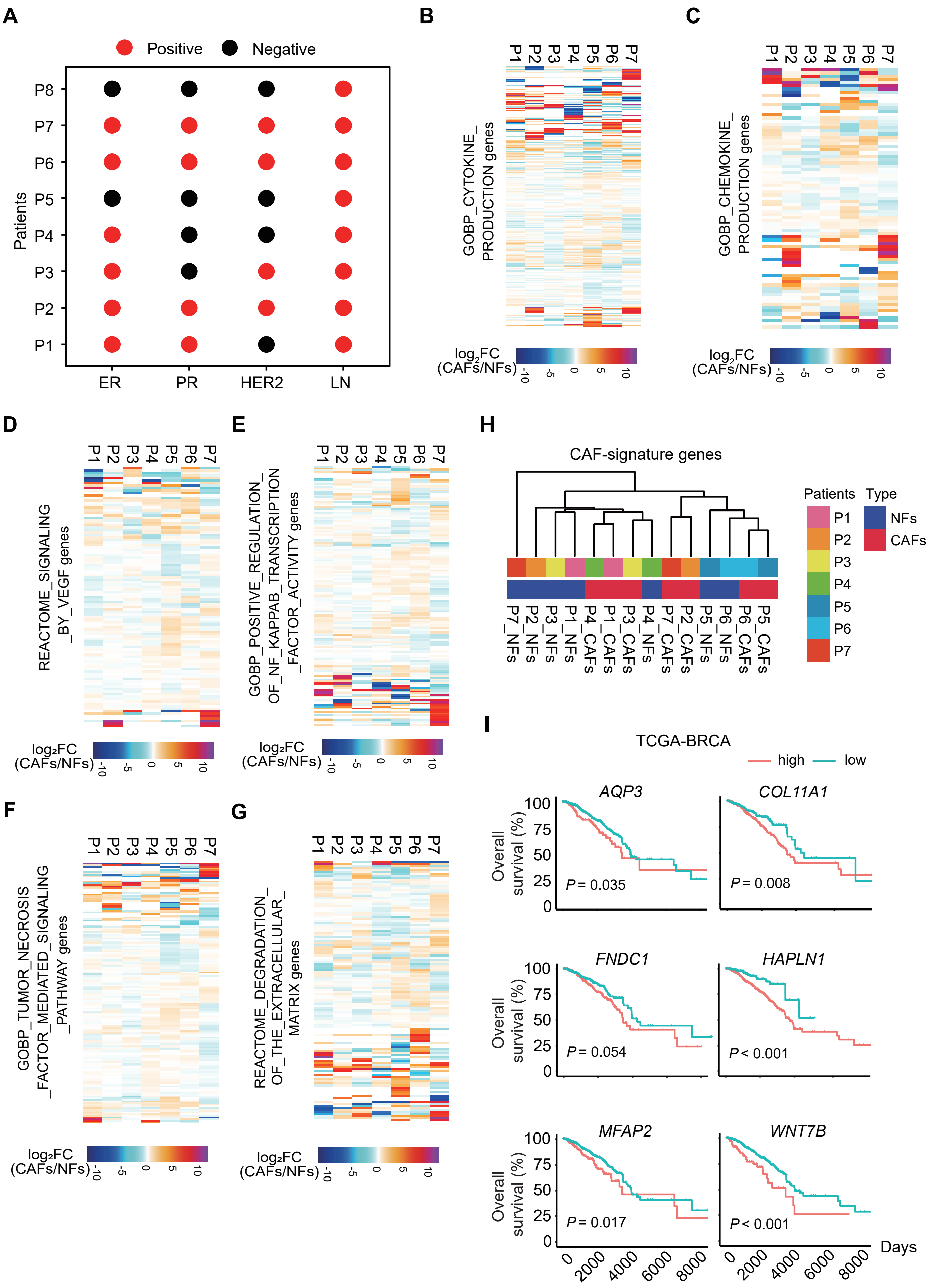

A
Positive
Negative
Patients
P8
P7
P6
P5
P4
P3
P2
P1
ER
PR
HER2
LN
B
GOBP_CYTOKINE_
PRODUCTION genes
log2FC
(CAFs/NFs)
C
GOBP_CHEMOKINE_
PRODUCTION genes
D
REACTOME_SIGNALING
_BY_VEGF genes
E
GOBP_POSITIVE_REGULATION_
OF_NF_KAPPAB_TRANSCRIPTION
_FACTOR_ACTIVITY genes
F
GOBP_TUMOR_NECROSIS
_FACTOR_MEDIATED_SIGNALING
_PATHWAY genes
G
REACTOME_DEGRADATION_
OF_THE_EXTRACELLULAR_
MATRIX genes
H
CAF-signature genes
Patients
Type
NFs
CAFs
P7_NFs
P2_NFs
P3_NFs
P1_NFs
P4_CAFs
P1_CAFs
P3_CAFs
P4_NFs
P7_CAFs
P2_CAFs
P5_NFs
P6_NFs
P6_CAFs
P5_CAFs
I
TCGA-BRCA
high
low
Overall survival (%)
AQP3
P = 0.035
COL11A1
P = 0.008
FNDC1
P = 0.054
HAPLN1
P < 0.001
MFAP2
P = 0.017
WNT7B
P < 0.001
Days

Figure S1. Transcriptional deregulation in metastasis associated CAFs. (**A**) Clinical pathologic information of breast cancer patients whose NFs and CAFs are used in our study. ER, estrogen receptor; PR, progesterone receptor; HER2, human epithelial growth fator receptor 2; LN, lymph node metastasis. (**B-G**) Using RNA-seq data in seven pairs of NFs and CAFs, heatmaps show $\log_2$ transformed fold change (CAFs/paired NFs) in mRNA levels of individual genes in designated gene sets. (**H**) Unsupervised hierarchical clustering of the expression profiles of CAF-signature genes in each designated sample. (**I**) Kaplan-Meier survival curves for correlation between mRNA expression levels of indicated genes and overall survival of breast cancer patients in the TCGA RNA-seq dataset. *P* values were determined by log-rank test.

Figure S2

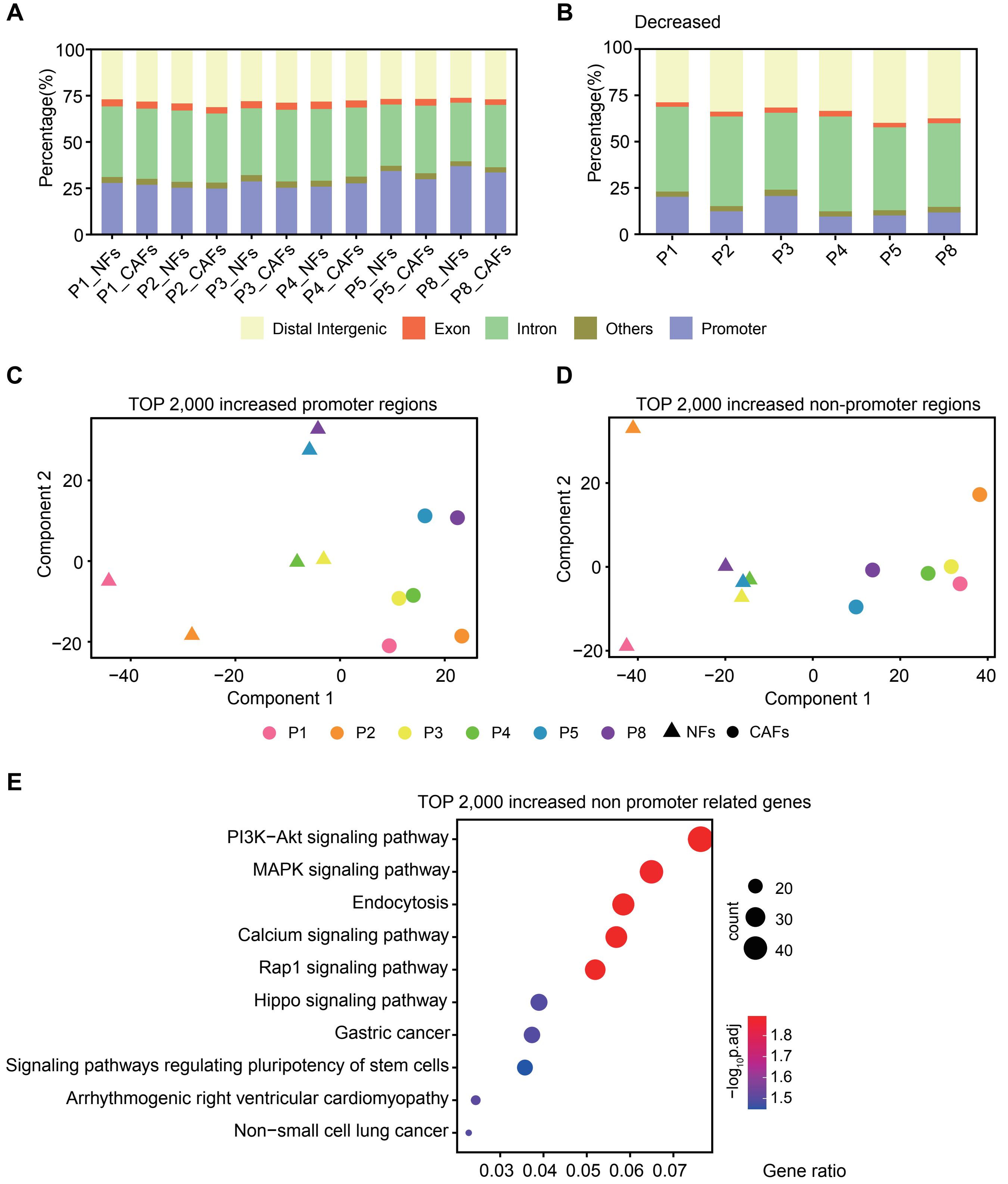

Figure S2. Regions with increased H3K27ac enrichment distinguish between NFs and CAFs. (**A**) Genomic distribution of peaks with H3K27ac enrichment in six pairs of NFs and CAFs. (**B**) Genomic distribution of peaks with decreased H3K27ac enrichment. (**C-D**) PCA plot for TOP 2,000 promoter regions (**C**) and non-promoter regions (**D**) with most increased H3K27ac enrichment. (**E**) Highly enriched KEGG pathways of nearest genes of TOP 2,000 increased non-promoter regions are shown.

Figure S3

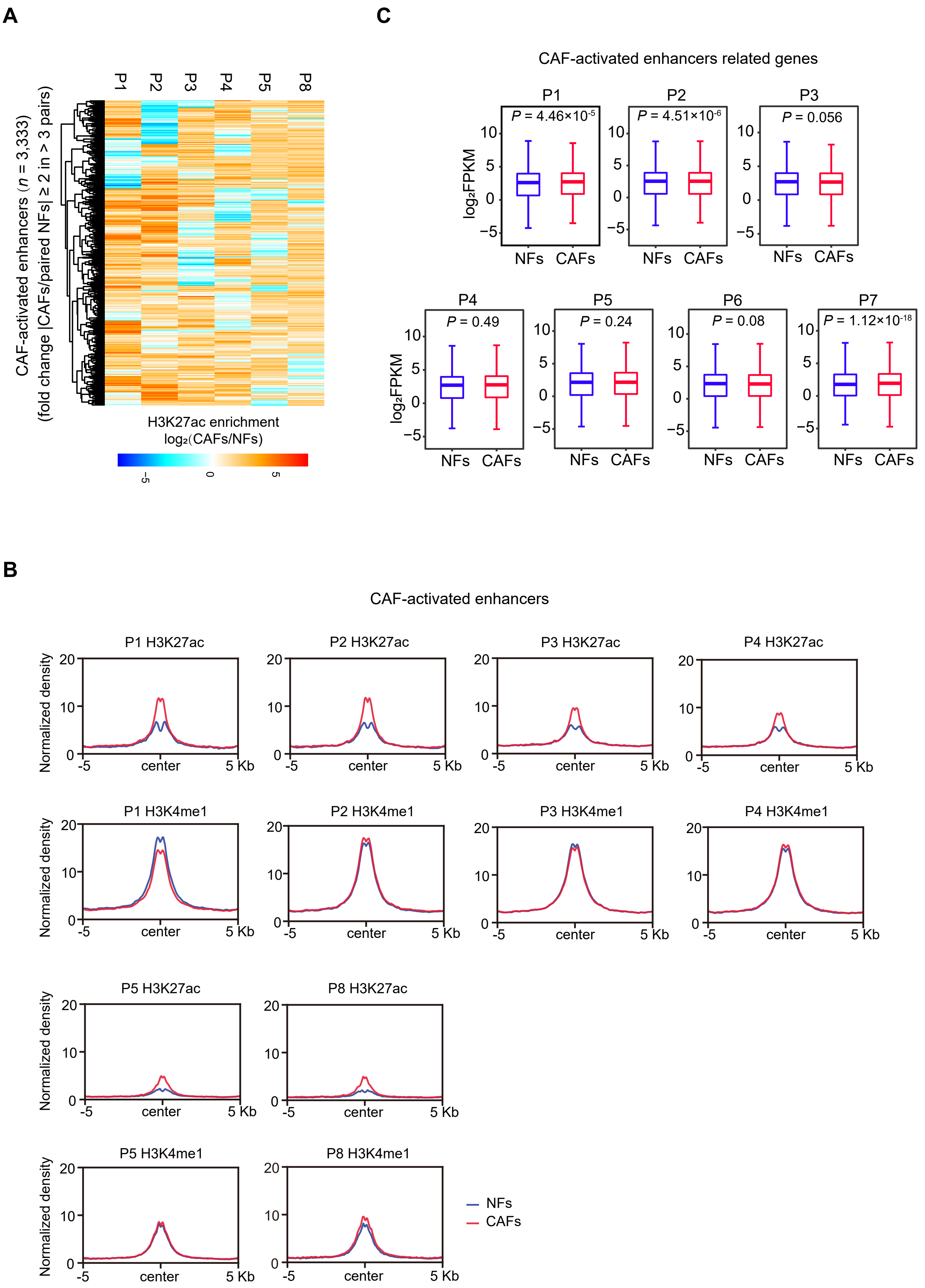

A
C
CAF-activated enhancers (n = 3,333)
(fold change |CAFs/paired NFs| ≥ 2 in > 3 pairs)
P1
P2
P3
P4
P5
P8
H3K27ac enrichment
log₂(CAFs/NFs)
−5
0
5
CAF-activated enhancers related genes
P1
P = 4.46×10⁻⁵
P2
P = 4.51×10⁻⁶
P3
P = 0.056
P4
P = 0.49
P5
P = 0.24
P6
P = 0.08
P7
P = 1.12×10⁻¹⁸
log₂FPKM
NFs
CAFs
B
CAF-activated enhancers
P1 H3K27ac
P2 H3K27ac
P3 H3K27ac
P4 H3K27ac
P1 H3K4me1
P2 H3K4me1
P3 H3K4me1
P4 H3K4me1
P5 H3K27ac
P8 H3K27ac
P5 H3K4me1
P8 H3K4me1
Normalized density
-5
center
5 Kb
NFs
CAFs

Figure S3. CAF-activated enhancers in CAFs versus NFs. (**A**) Heatmap shows H3K27ac enrichment by $\log_2$ (CAFs/paired NFs) on the CAF-activated enhancers. (**B**) Average profiles of H3K27ac and H3K4me1 CUT&Tag-seq signals across a genomic window of ± 5,000 bp surrounding the center of CAF-activated enhancers. (**C**) Boxplots to compare the Log_2 transformed mRNA expression levels of CAF-activated enhancers nearest genes in NFs and CAFs. The NFs and CAFs were isolated from Patient 1-7 (P1-P7) respectively. *P* values were determined by one-sided paired *t* test.

Figure S4

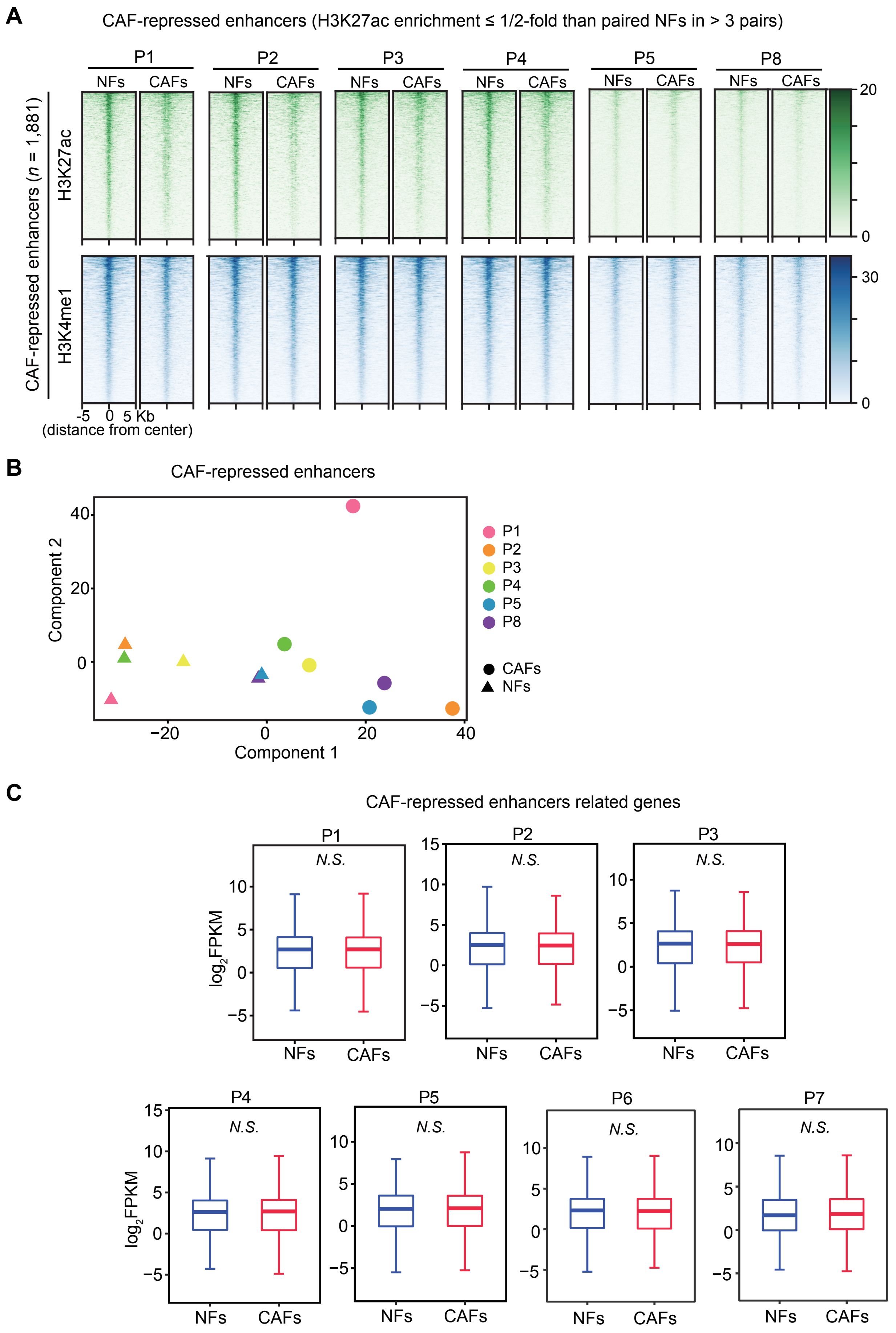
A
CAF-repressed enhancers (H3K27ac enrichment ≤ 1/2-fold than paired NFs in > 3 pairs)
P1
P2
P3
P4
P5
P8
NFs
CAFs
CAF-repressed enhancers (n = 1,881)
H3K27ac
H3K4me1
20
0
30
0
-5 0 5 Kb
(distance from center)
B
CAF-repressed enhancers
Component 2
Component 1
P1
P2
P3
P4
P5
P8
CAFs
NFs
C
CAF-repressed enhancers related genes
P1
P2
P3
P4
P5
P6
P7
N.S.
log2FPKM
NFs
CAFs

Figure S4. CAF-repressed enhancers in primary CAFs. (**A**) Heatmaps of H3K27ac and H3K4me1 in six pairs of NFs and paired CAFs across regions of ± 5,000 bp surrounding the center of CAF-repressed enhancers. (**B**) PCA plot of CAF-repressed enhancers for H3K27ac CUT&Tag-seq signals in each sample. (**C**) Boxplots to compare the Log_2 transformed mRNA expression levels of CAF-repressed enhancers nearest genes in NFs and CAFs. The NFs and CAFs were isolated from Patient 1-7 (P1-P7) respectively. *P* values were determined by one-sided paired *t* test. *N.S.*, non-significant.

Figure S5

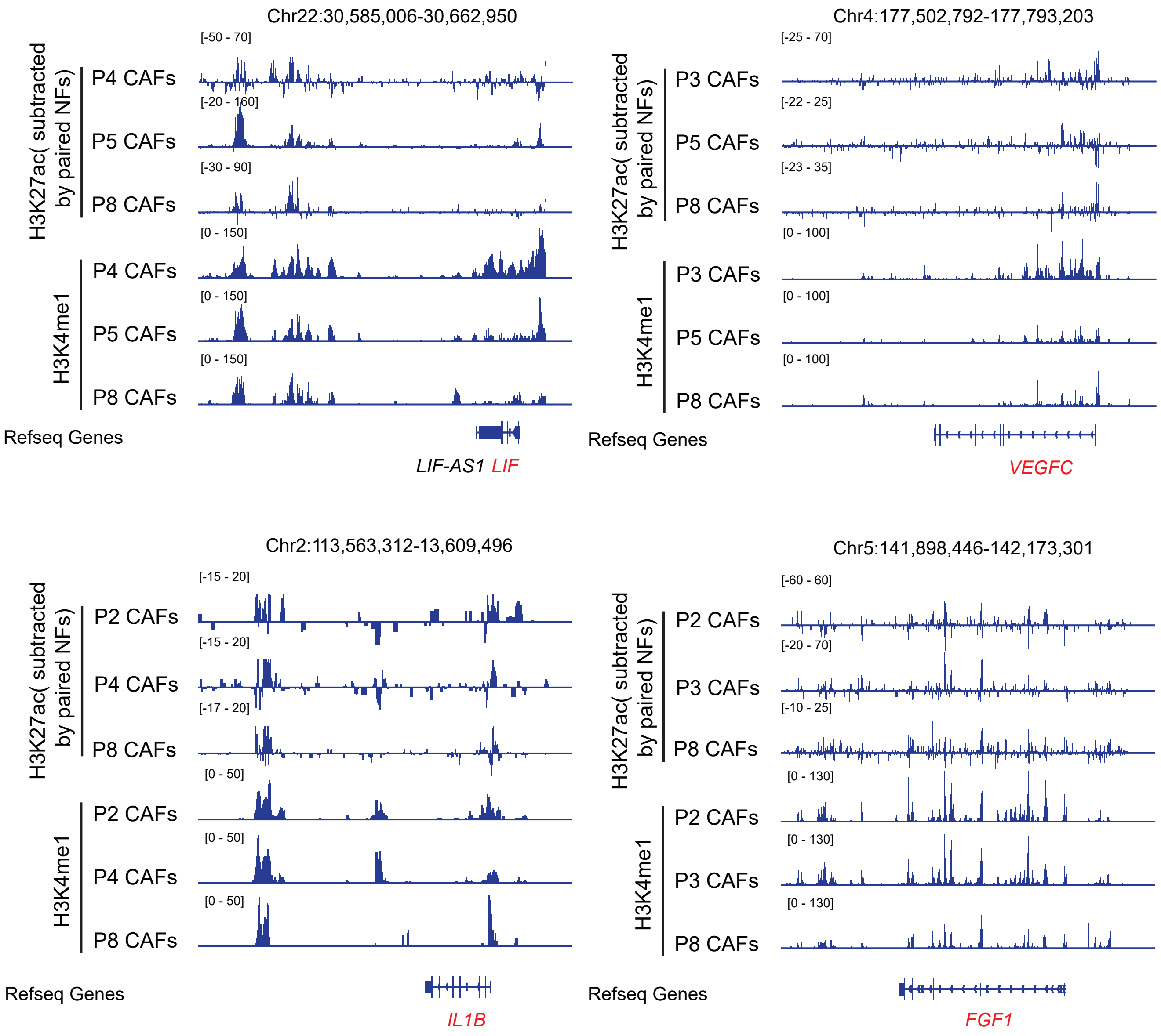

Figure S5. Increased H3K27ac enrichment at CAF-activated enhancers in CAFs compared with NFs. Representative genomic snapshots to compare the H3K27ac CUT&Tag-seq signals of two histone modifications at designated CAF-activated enhancers. The subtracted signals (CAFs-paired NFs) are shown for H3K27ac while the H3K4me1 signals are directly shown in CAFs.

Figure S6

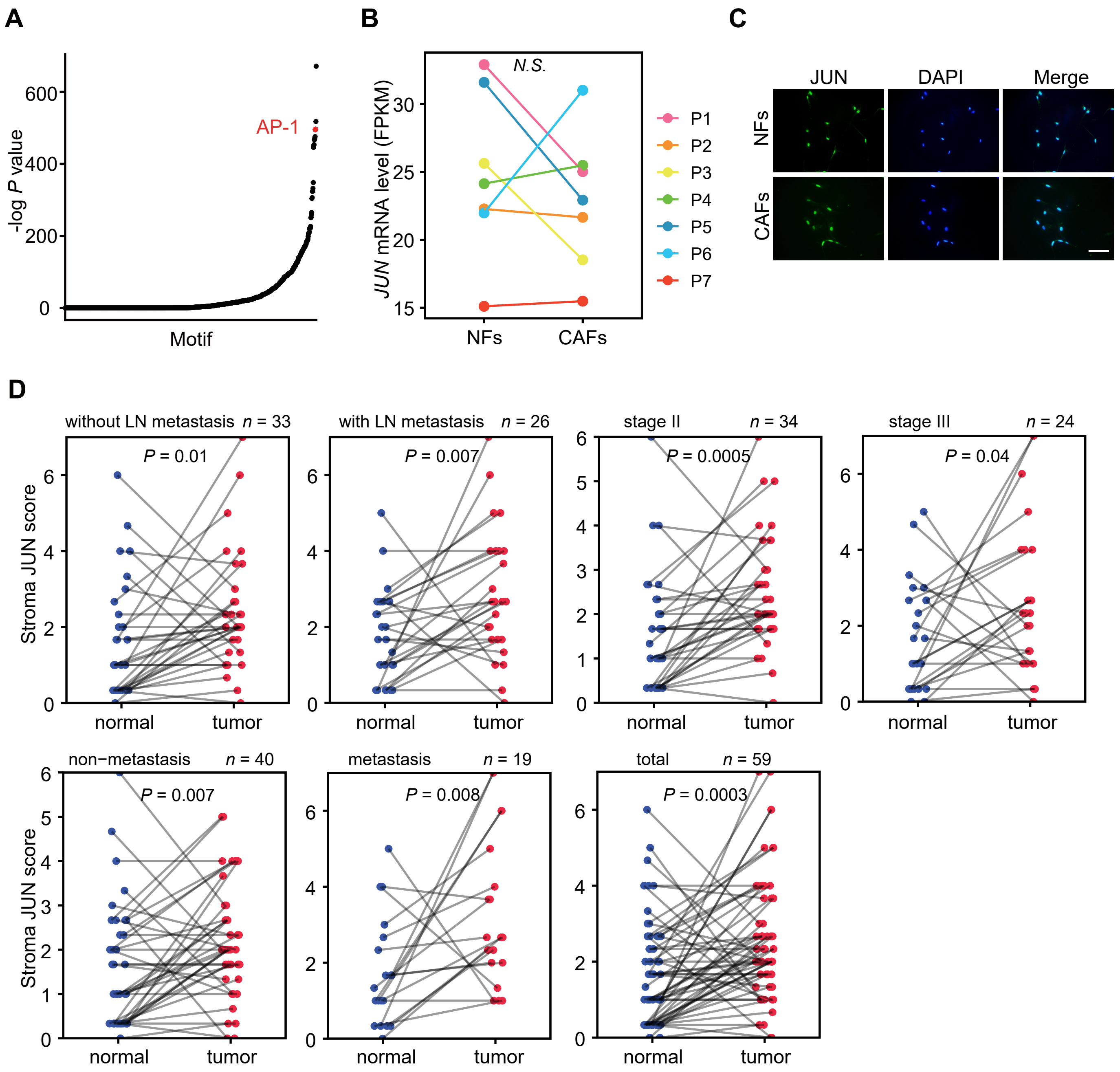

Figure S6. Total protein levels of JUN in breast cancer stroma. (**A**) Motif analysis of regions of ± 300 bp surrounding CAF-activated enhancers center using HOMER software. The data is shown in the order of -log *P* value. (**B**) *JUN* mRNA levels are compared in NFs and CAFs, according to the RNA-seq data. *P* value was determined by one-sided Wilcoxon signed rank exact test. *N.S.*, non-significant. (**C**) JUN (green) and DAPI (blue) staining of NFs and paired CAFs. Scale bars, 100 μm. (**D**) Stroma immunostaining scores of JUN in para-cancerous tissues and paired tumor tissues of indicated samples are shown as line plot. *P* values were determined by one-sided paired *t* test.

Figure S7

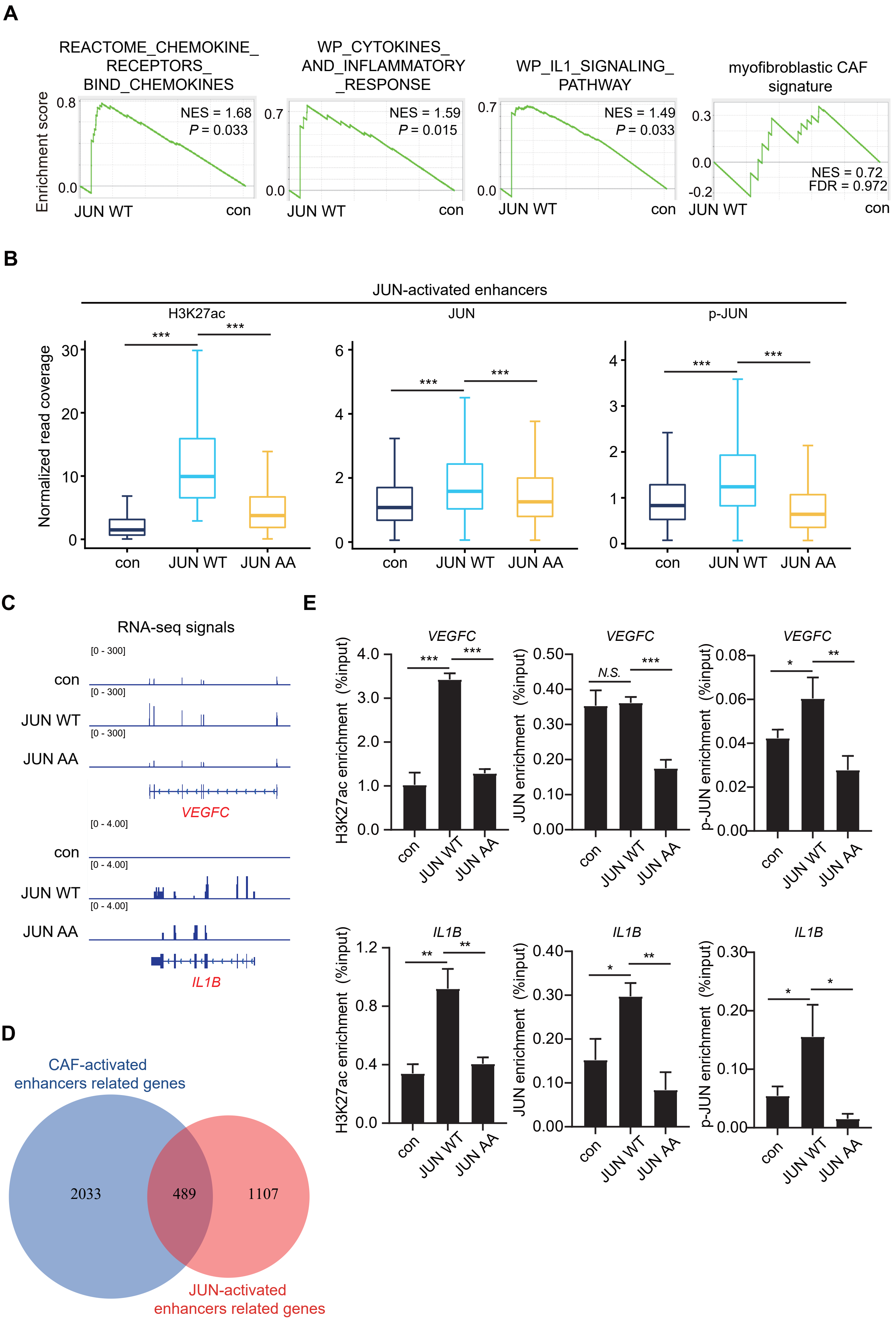

A
REACTOME_CHEMOKINE_RECEPTORS_BIND_CHEMOKINES
NES = 1.68
P = 0.033
WP_CYTOKINES_AND_INFLAMMATORY_RESPONSE
NES = 1.59
P = 0.015
WP_IL1_SIGNALING_PATHWAY
NES = 1.49
P = 0.033
myofibroblastic CAF signature
NES = 0.72
FDR = 0.972
Enrichment score
JUN WT
con
B
JUN-activated enhancers
H3K27ac
JUN
p-JUN
Normalized read coverage
con
JUN WT
JUN AA
C
RNA-seq signals
[0 - 300]
[0 - 4.00]
VEGFC
IL1B
D
CAF-activated enhancers related genes
JUN-activated enhancers related genes
2033
489
1107
E
VEGFC
IL1B
H3K27ac enrichment (%input)
JUN enrichment (%input)
p-JUN enrichment (%input)
N.S.

Figure S7. Phosphorylated JUN drives enhancer activation and gene expression. (**A**) GSEA for the indicated signatures based on the RNA-seq data of MRC5 overexpressing JUN WT and control. (**B**) Normalized read coverages of H3K27ac, JUN and p-JUN ChIP-seq signals at JUN-activated enhancers are shown as boxplots. *P* value was determined by two-sided paired *t* test. ***, $P < 0.001$. (**C**) Tracks of RNA-seq signals of designated genes in designated groups of cells. (**D**) Venn diagram showing the overlap between primary CAF-activated enhancers associated genes and JUN-activated enhancers associated genes. (**E**) ChIP-qPCR analysis of H3K27ac, JUN and p-JUN enrichment at the activated enhancers associated with *VEGFC* and *IL1B*. Data are presented as mean ± SD of three independent biological replicates. *P* value was determined by two-sided unpaired *t* test. *, $P < 0.05$; **, $P < 0.01$; ***, $P < 0.001$. *N.S.*, non-significant.

Figure S8

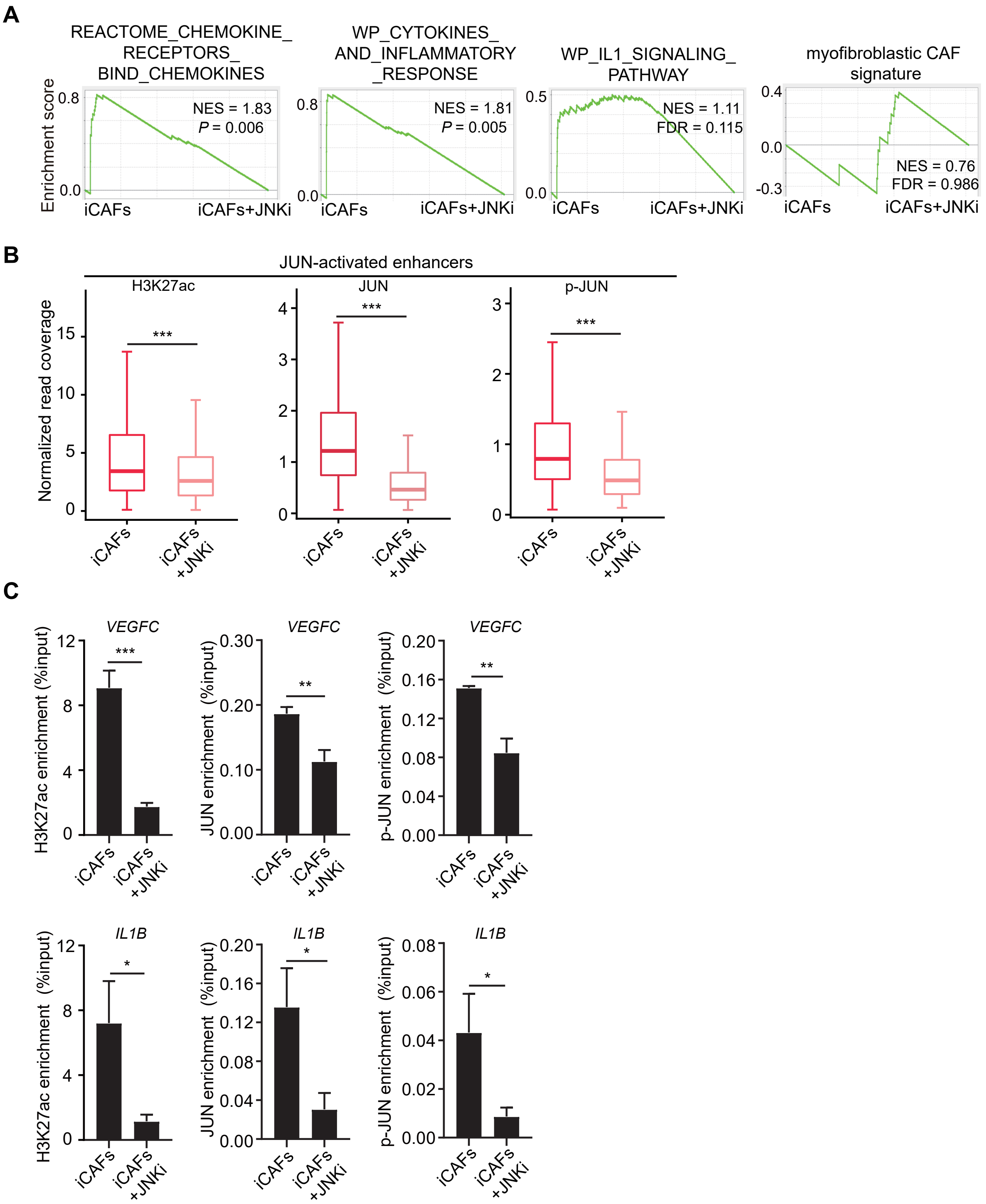
A
REACTOME_CHEMOKINE_RECEPTORS_BIND_CHEMOKINES
NES = 1.83
P = 0.006
WP_CYTOKINES_AND_INFLAMMATORY_RESPONSE
NES = 1.81
P = 0.005
WP_IL1_SIGNALING_PATHWAY
NES = 1.11
FDR = 0.115
myofibroblastic CAF signature
NES = 0.76
FDR = 0.986
Enrichment score
iCAFs
iCAFs+JNKi
B
JUN-activated enhancers
H3K27ac
JUN
p-JUN
Normalized read coverage
***
iCAFs
iCAFs +JNKi
C
VEGFC
IL1B
H3K27ac enrichment (%input)
JUN enrichment (%input)
p-JUN enrichment (%input)
***
**
*

Figure S8. Phosphorylated JUN is required for maintenance of the activated enhancers. (**A**) GSEA for the indicated signatures based on the RNA-seq data of iCAFs and iCAFs+JNKi. (**B**) Normalized read coverages of H3K27ac, JUN and p-JUN ChIP-seq signals at JUN-activated enhancers are shown as boxplots. *P* value was determined by two-sided paired *t* test. ***, $P < 0.001$. (**C**) ChIP-qPCR analysis of H3K27ac, JUN and p-JUN enrichment at the active enhancers associated with *VEGFC* and *IL1B*. Data are presented as mean ± SD of three independent biological replicates. *P* values were determined by two-sided unpaired *t* test. *, $P < 0.05$; **, $P < 0.01$; ***, $P < 0.001$.

Figure S9

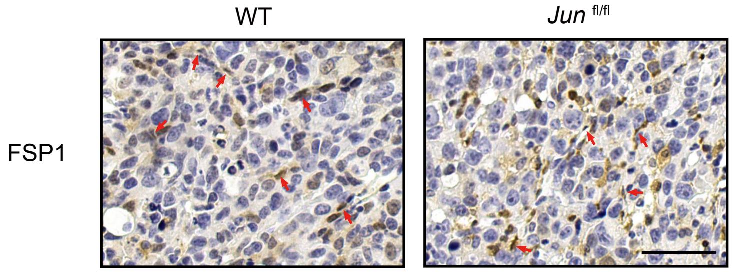

Figure S9. FSP1 is extensively expressed in CAF-like cells inside the tumors. Immunostaining of FSP-1 in allografted tumors. Fibroblasts are labeled using red arrows. Scale bars, 50 μm.

Table S1. Clinical features of patients involved in this study.

Patient	Gender	Age	Pathological type	Stage	LN	ER	PR	HER2	Ki67	Tumor size
P1	female	65	invasive ductal carcinoma	II	8/21	+	+	negative	10%	1.7 cm × 1.5 cm × 1.1 cm
P2	female	54	invasive ductal carcinoma	II	1/15	+	+	positive	30%	1.5 cm × 1 cm × 1 cm
P3	female	44	invasive ductal carcinoma	II	13/19	+	-	positive	30%	1.5 cm × 1.5 cm × 1 cm
P4	female	63	invasive ductal carcinoma	II	5/17	+	-	negative	5%	2 cm × 1.5 cm × 1.2 cm
P5	female	40	invasive ductal carcinoma	III	4/21	-	-	negative	60%	6 cm × 5 cm × 2 cm
P6	female	44	invasive ductal carcinoma	III	1/16	+++	+++	positive	30%	2 cm × 1.5 cm × 1.1 cm
P7	female	65	invasive ductal carcinoma	I	1/18	+	+	positive	10%	1.8 cm × 1 cm × 1 cm
P8	female	46	invasive ductal carcinoma	III	1/32	-	-	negative	15%	3 cm × 2.5 cm × 1.5 cm

Table S2 Alignment summary of CUT&Tag sequencing data.

Sample	Total reads	Overall alignment rate	Unique aligned reads	Unique alignment rate
P1_NFs_H3K27ac	22643810	88.00%	16330735	72.12%
P1_NFs_H3K4me1	20761984	92.39%	16225920	78.15%
P1_CAFs_H3K27ac	20467107	93.51%	15674005	76.58%
P1_CAFs_H3K4me1	34688571	93.69%	25216460	72.69%
P2_NFs_H3K27ac	23145607	93.15%	17616486	76.11%
P2_NFs_H3K4me1	18961157	95.78%	15207156	80.20%
P2_CAFs_H3K27ac	19761418	90.46%	14411438	72.93%
P2_CAFs_H3K4me1	23394666	95.23%	18560383	79.34%
P3_NFs_H3K27ac	20357350	93.08%	15084971	74.10%
P3_NFs_H3K4me1	22847906	95.38%	18272793	79.98%
P3_CAFs_H3K27ac	20653417	97.37%	16030644	77.62%
P3_CAFs_H3K4me1	23057999	94.78%	18030640	78.20%
P4_NFs_H3K27ac	19371281	96.15%	14475316	74.73%
P4_NFs_H3K4me1	25604433	94.50%	20054609	78.32%
P4_CAFs_H3K27ac	23963310	95.57%	18005737	75.14%
P4_CAFs_H3K4me1	24816689	94.05%	19431091	78.30%
P5_NFs_H3K27ac	22888257	55.39%	8299480	36.26%
P5_NFs_H3K4me1	32699042	57.60%	15042898	46.00%
P5_CAFs_H3K27ac	28769583	53.35%	9882750	34.35%
P5_CAFs_H3K4me1	30779098	60.26%	14479107	47.04%
P8_NFs_H3K27ac	32841930	43.71%	9449082	28.77%
P8_NFs_H3K4me1	12198827	60.62%	5768923	47.29%
P8_CAFs_H3K27ac	27287888	46.81%	9166586	33.59%
P8_CAFs_H3K4me1	13777194	61.56%	7004668	50.84%
